# Immune Infiltration-Related ceRNA Network Revealing Potential Biomarkers for Prognosis of Head and Neck Squamous Cell Carcinoma

**DOI:** 10.1155/2022/1014347

**Published:** 2022-09-02

**Authors:** Shuai Zhao, Mengle Peng, Zhongquan Wang, Jingjing Cao, Xinyu Zhang, Ruijing Yu, Tao Huang, Wenping Lian

**Affiliations:** ^1^Department of Clinical Laboratory, Henan Provincial Third People's Hospital, Zhengzhou, 450006 Henan, China; ^2^Department of Basis Medicine, Henan Medical College, Zhengzhou, 451191 Henan, China; ^3^Department of Medical Affair, Henan Provincial Third People's Hospital, Zhengzhou, 450006 Henan, China; ^4^Medical School, Huanghe Science and Technology University, 666 Zi Jing Shan Road, Zhengzhou, 450000 Henan, China

## Abstract

**Background:**

Head and neck squamous cell carcinoma (HNSCC) is a frequently lethal malignancy, and the mortality is considerably high. The tumor microenvironment (TME) has been identified as a critical participation in cancer development, treatment, and prognosis. However, competing endogenous RNA (ceRNA) networks grouping with immune/stromal scores of HNSCC patients need to be further illustrated. Therefore, our study aimed to provide clues for searching promising prognostic markers of TME in HNSCC.

**Materials and Methods:**

ESTIMATE algorithm was used to calculate immune scores and stromal scores of the enrolled HNSCC patients. Differentially expressed genes (DEGs), lncRNAs (DELs), and miRNAs (DEMs) were identified by comparing the expression difference between high and low immune/stromal scores. Then, a ceRNA network and protein-protein interaction (PPI) network were constructed for selecting hub regulators. In addition, survival analysis was performed to access the association between immune scores, stromal scores, and differentially expressed RNAs in the ceRNA network and the overall survival (OS) of HNSCC patients. Then, the GSE65858 datasets from Gene Expression Omnibus (GEO) database was used for verification. At last, the difference between the clinical characteristics and immune cell infiltration in different expression groups of IL10RA, PRF1, and IL2RA was analyzed.

**Results:**

Survival analysis showed a better OS in the high immune score group, and then we constructed a ceRNA network composed of 97 DEGs, 79 DELs and 22 DEMs. Within the ceRNA network, FOXP3, IL10RA, STAT5A, PRF1, IL2RA, miR-148a-3p, miR-3065-3p, and lncRNAs, including CXCR2P1, HNRNPA1P21, CTA-384D8.36, and IGHV1OR15-2, were closely correlated with the OS of HNSCC patients. Especially, using the data from GSE65858, we successfully verified that IL10RA, PRF1, and IL2RA were not only significantly upregulated in patients high immune scores, but also their high expressions were associated with longer survival time. In addition, stratified analysis showed that PRF1 and IL2RA might be involved in the mechanism of tumor progress.

**Conclusion:**

In conclusion, we constructed a ceRNA network related to the TME of HNSCC, which provides candidates for therapeutic intervention and prognosis evaluation.

## 1. Introduction

Head and neck cancer is a frequently lethal malignancy, with approximately 800,000 new cases every year [1, 2]. The head and neck squamous cell carcinoma (HNSCC) subtype accounts for almost 95% of head and neck cancers [3]. Despite significant advances in different therapy methods, such as chemotherapy, monoclonal antibody therapies, immunotherapy, and cytokine therapy [4, 5], the mortality of HNSCC is considerably high, mainly due to the heterogeneity, aggressiveness, and late diagnosis of HNSCC [6]. Thus, studies on the molecular mechanisms of HNSCC to discover effective biomarkers and targeted therapy to precisely predict prognosis are necessary.

Currently, the tumor microenvironment (TME), consisting of extracellular matrix, stromal cells, and tumor-infiltrating immune cells, is known to be involved in cancer development, distant metastasis, and immune escape [7]. Bidirectional communication between tumor cells and their microenvironment causes the continual change over the evolution of tumors, and various tumor-secreted factors, such as oncoproteins and oncopeptides, RNA species (such as mRNAs, miRNAs, and lncRNAs), lipids, and DNA fragments, are known to participate in this communication [8, 9]. The biological alterations present in the TME provide target molecules that facilitate prognosis evaluation and anticancer therapies [9, 10]. LncRNAs and miRNAs are common types of noncoding RNAs that play multiple roles in normal physiology and pathological processes [11]. The competing endogenous RNA (ceRNA) hypothesis figures that RNA transcripts communicate with each other by competing for shared miRNAs, which act as a widespread form of posttranscriptional regulation of gene expression [12, 13]. So far, several studies have investigated prognostic value of ceRNA networks in HNSCC, and the differential expressed RNAs involved were all obtained from comparisons of HNSCC cases and normal samples. For instance, Pan et al. constructed a ceRNA network in HNSCC patients and identified some miRNAs (hsa-mir-99a, hsa-mir-337, and hsa-mir-137) and mRNAs (NOSTRIN, TIMP4, GRB14, HOXB9, CELSR3, and ADGRD2) that might be prognostic biomarkers in HNSCC [14]. Zhou et al. constructed a ceRNA-related signature and speculated that the interactions among KCNQ1OT1, hsa-miR-148a-3p, ITGA5, and naive B cells might closely correlate with the initiation and progression of HNSCC [15]. Wang et al. investigated the role of the immune microenvironment in the development and prognosis of HPV-negative HNSCC tumors by constructing a ceRNA network [16]. Yang et al. identified five lncRNAs (MIR4435-2HG, CASC9, LINC01980, STARD4-AS1, and MIR99AHG) with remarkable association with OS of HNSCC patients and one lncRNA (PART1) with a superior performance in differentiating HNSCC tissues from non-HNSCC normal tissues [17]. However, ceRNA networks grouping with immune/stromal scores of HNSCC patients need to be further illustrated.

In this study, we firstly divided HNSCC patients into two groups according to the immune/stromal scores with the ESTIMATE algorithm. Differentially expressed genes (DEGs), lncRNAs (DELs), and miRNAs (DEMs) were identified between the high- and low-score groups. Then, Kaplan-Meier survival analysis was performed to explore the relationship between immune/stromal scores and overall survival (OS). In light of the better OS of patients with high immune scores, a ceRNA network was constructed using the DEGs, DELs, and DEMs from the high and low immune score groups. In addition, a PPI network of DEGs was constructed to select hub genes, and survival analysis was performed to evaluate the prognostic roles of these RNAs included in the ceRNA network. Furthermore, the different expression and prognostic value of the survival-related RNAs were verified using the GSE65858 dataset from Gene Expression Omnibus (GEO) database. Finally, analysis of the clinical relevance and immune cell infiltration for IL10RA, PRF1, and IL2RA were conducted.

## 2. Materials and Methods

### 2.1. Data Acquisition

The RNA-sequencing (FPKM) and clinical characteristics of 468 HNSCC patients were obtained from the TCGA Database (https://tcga-data.nci.nih.gov/tcga/). Patients with other malignant tumors were excluded from our study, and samples that possessed the mRNA, miRNA, and lncRNA expression data simultaneously were included. One HNSCC cohort of GEO database (GSE65858) with 270 HNSCC patients was used for validation.

### 2.2. Stromal and Immune Scores Based on the ESTIMATE Algorithm

Immune scores and stromal scores were calculated by using the estimate R package (version 4.0.3) [18]. According to the median score of infiltrating immune/stromal cells, HNSCC patients were divided into two groups. Furthermore, Kaplan-Meier survival analysis was performed to illustrate the relationship between the OS and the immune/stromal scores of HNSCC patients using the survival package of R.

### 2.3. Identification of DEGs, DELs, and DEMs

The DEGs, DELs, and DEMs between the two groups were determined with the limma package of R. The DEGs and DEMs were selected with *P* < 0.05, false discovery rate (FDR) < 0.05, and log2|fold change (FC)| > 1.5. When determining the DELs, *P* < 0.05, FDR < 0.05, and log2|FC| > 1.2 were used as cutoff values because there were so few candidate lncRNAs. Furthermore, the heatmap packages were applied to generate the heatmaps of DEGs, DELs, and DEMs.

### 2.4. Functional Analysis of DEGs

Gene ontology (GO) categories by molecular function (MF) and cellular component (CC) and biological process (BP), as well as Kyoto Encyclopedia of Genes and Genomes (KEGG) pathway enrichment analyses of DEGs were conducted by using ggplot2, enrichplot, and clusterProfiler package of R. *P* values less than 0.05 were considered significantly enriched.

### 2.5. ceRNA Network Construction

Considering the prognostic relevance of immune/stromal scores in HNSCC patients, we selected the groups with a better *P* value for further analysis. MiRanda, TargetScan, and miRWalk were used to predict miRNA-mRNA interactions, and miRanda and PITA were used to predict miRNA-lncRNA interactions. Then, the intersection was taken between the target mRNAs/lncRNAs and the previously identified DEGs/DELs. Furthermore, DEMs that negatively regulated the expression of DEL and DEGs were retained to construct the ceRNA network and visualized via Cytoscape v3.8.0.

### 2.6. PPI Network and Survival Analysis

By using the STRING database, a PPI network of DEGs included in the ceRNA network was constructed and then visualized with Cytoscape. And Kaplan-Meier analysis was performed to investigate the relationship between the expression of DEGs, DELs, and DEMs in the ceRNA network and OS of HNSCC patients. *P* < 0.05 was recognized as a statistically significant difference.

### 2.7. Analysis of the Clinical Relevance and Immune Cell Infiltration for IL10RA, PRF1, and IL2RA

Based on clinical characteristics (age, sex, tumor stage, TNM stage, grade, smoking, radiation, and therapy), HNSCC patients were stratified into distinct subgroups. A Chi-square test was performed to determine the difference in clinical characteristics between different expression groups of IL10RA, PRF1, and IL2RA. Besides, QUANTISEQ (https://icbi.i-med.ac.at/software/quantiseq/doc/) was employed to access difference in immune cells infiltration.

## 3. Results

### 3.1. Clinical Characteristics of HNSCC Patients

468 HNSCC patients were eventually enrolled in our study, and the clinicopathological characteristics obtained from the TCGA database were summarized in Table S1. The age ranged from 19 to 90 years, and 346 (73.9%) were male and 122 (26.1%) were female. The median survival time was 625 days, ranged from 2 to 6417 days.

### 3.2. Immune Scores and Stromal Scores of HNSCC Patients

Immune scores and stromal scores were used to infer the level of infiltrating stromal and immune cells in tumor tissues. The 468 HNSCC patients were categorized into lower and upper halves based on the median immune/stromal scores. And the immune scores ranged from − 1088.39 to 2912.77, the stromal scores ranged from − 2092.21 to 1989.27 (Table S2).

**Figure 1:**
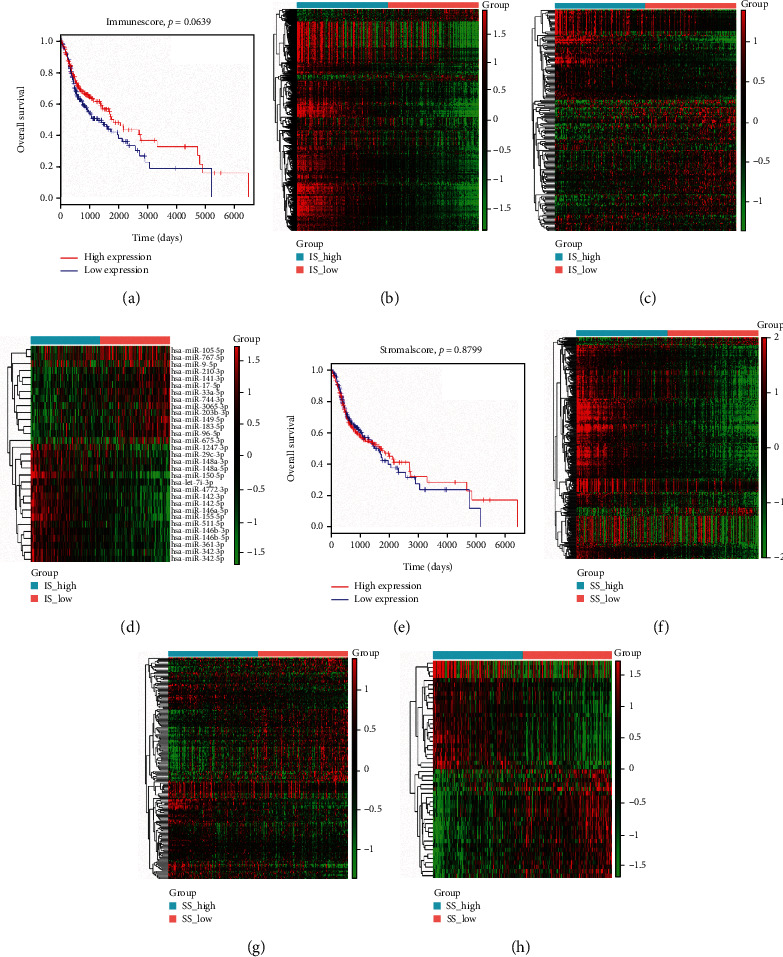
Gene expression profiles and survival analysis based on immune scores and stromal scores. (a) Kaplan-Meier survival curves of high (red line) and low (blue line) immune scores. Immune scores for the heatmaps of (b) DEGs, (c) DELs, and (d) DEMs. (e) Kaplan-Meier survival curves of high (red line) and low (blue line) stromal scores. Stromal scores for the heatmaps (f) DEGs, (g) DELs, and (h) DEMs.

Kaplan-Meier survival curves showed that patients with higher stromal scores and immune scores had longer survival times than those with lower scores, although these differences in survival were not statistically significant (*P* = 0.0639 and *P* = 0.8799, respectively) (Figures 1(a) and 1(e)).

### 3.3. Identification of DEGs, DELs, and DEMs

A total of 569 DEGs, 185 DELs, and 31 DEMs were identified between the high and low immune score groups, and 384 DEGs, 186 DELs, and 50 DEMs were obtained between the high and low stromal score groups. Heatmaps of the DEGs, DELs, and DEMs in these two comparisons were generated and are shown in Figure 1.

### 3.4. GO and KEGG Enrichment Analyses of the DEGs

**Figure 2:**
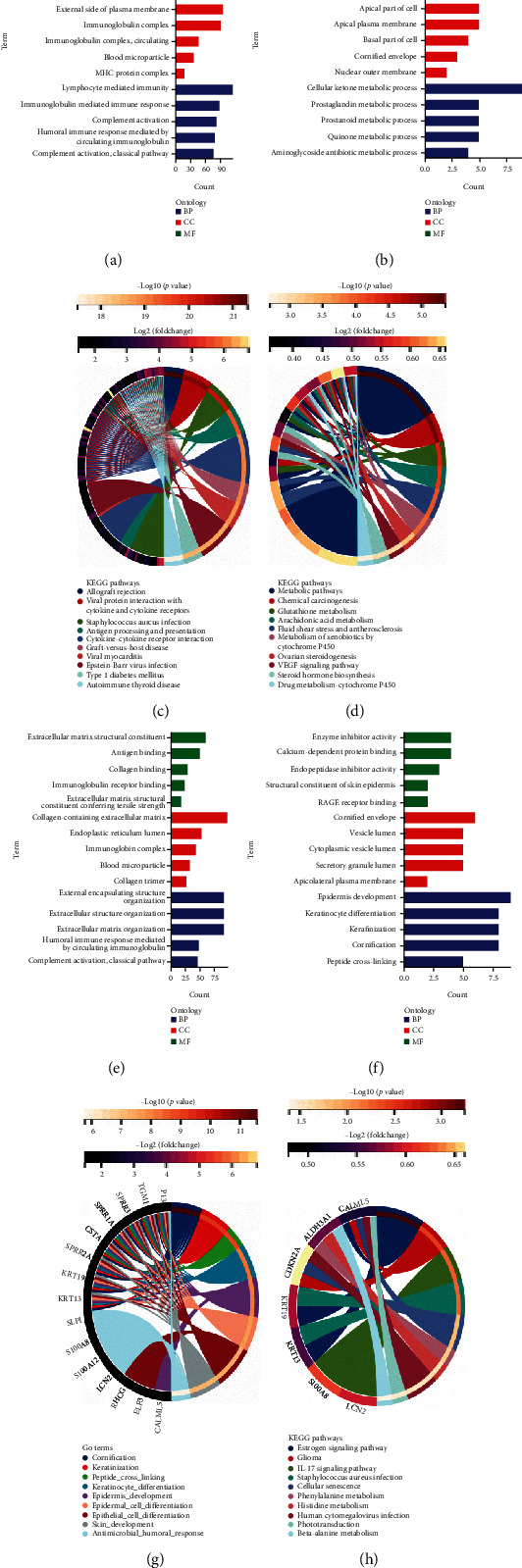
Gene expression profiles based on GO and KEGG for immune scores and stromal scores. Immune scores for GO terms for (a) upregulated DEGs and (b) downregulated DEGs. Enrichment of pathways for (c) upregulated DEGs and (d) downregulated DEGs. Stromal scores for GO terms for (e) upregulated DEGs and (f) downregulated DEGs. Enrichment of pathways for (g) upregulated DEGs and (h) downregulated DEGs.

Enrichment analysis of the 953 DEGs identified in the previous section was performed to reveal their potential functions. GO terms of upregulated DEGs in the immune score groups included “antigen binding,” “external side of plasma membrane,” and “lymphocyte mediated immunity” in MF, CC, and BP, respectively. For downregulated DEGs, the top GO terms included “aldo-keto redustase (NADP) activity” in MF, “apical part of cell” in CC, and “cellular ketone metabolic process” in BP. The enriched KEGG pathways of those upregulated DEGs were mainly involved in “allograft rejection” and “viral protein interaction with cytokine and cytokine receptor,” and the main KEGG term enriched by the downregulated DEGs was “metabolic pathways” (Figures 2(a)–2(d)).

In addition, the top GO terms of the upregulated DEGs in the stromal score groups included “extracellular matrix structural constituent,” “collagen-containing extracellular matrix,” and “external encapsulating structure organization” in MF, CC, and BP, respectively. For the downregulated DEGs, the top GO terms included “enzyme inhibitor activity” in MF, “cornified envelope” in CC, and “epidermis development” in BP. The KEGG pathways associated with the upregulated DEGs mainly involved pathways related to “cornification,” and the KEGG terms associated with the downregulated DEGs included “estrogen signaling pathway” (Figures 2(e)–2(h)).

### 3.5. ceRNA Network

**Figure 3:**
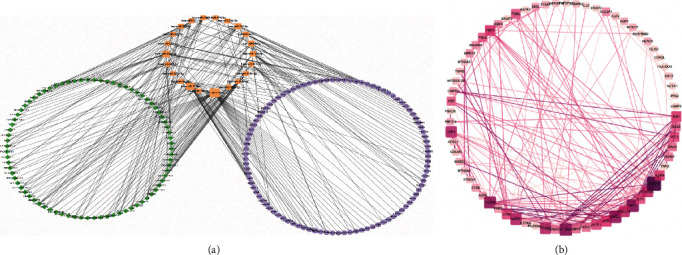
The ceRNA network and PPI network. (a) The ceRNA network. The diamond, rectangle, and oval shape represent DELs, DEMs, and DEGs, respectively. (b) The PPI network of DEGs. The lines indicate interactions between the RNAs.

There were no significant differences in OS between patients with high and low immune/stromal scores (*P* = 0.0639 and *P* = 0.8799), while it did not mean that the DEGs, DELs, and DEMs between the two groups had no prognostic values. Thus, we chose DEGs, DELs, and DEMs of immune score groups, which had a relatively better survival for the construction of ceRNA network (Figure 1(a)). Finally, the ceRNA network contained 926 edges composed of 97 DEGs, 79 DELs, and 22 DEMs were constructed (Figure 3(a)). Especially, hsa-miR-149-5p, has-miR-17-5p, hsa-miR-3065-3p, hsa-miR-767-5p, and hsa-miR-96-5p were the top 5 nodes, suggesting that they might be master regulators in the network.

### 3.6. PPI Network Construction and Survival Analysis

**Figure 4:**
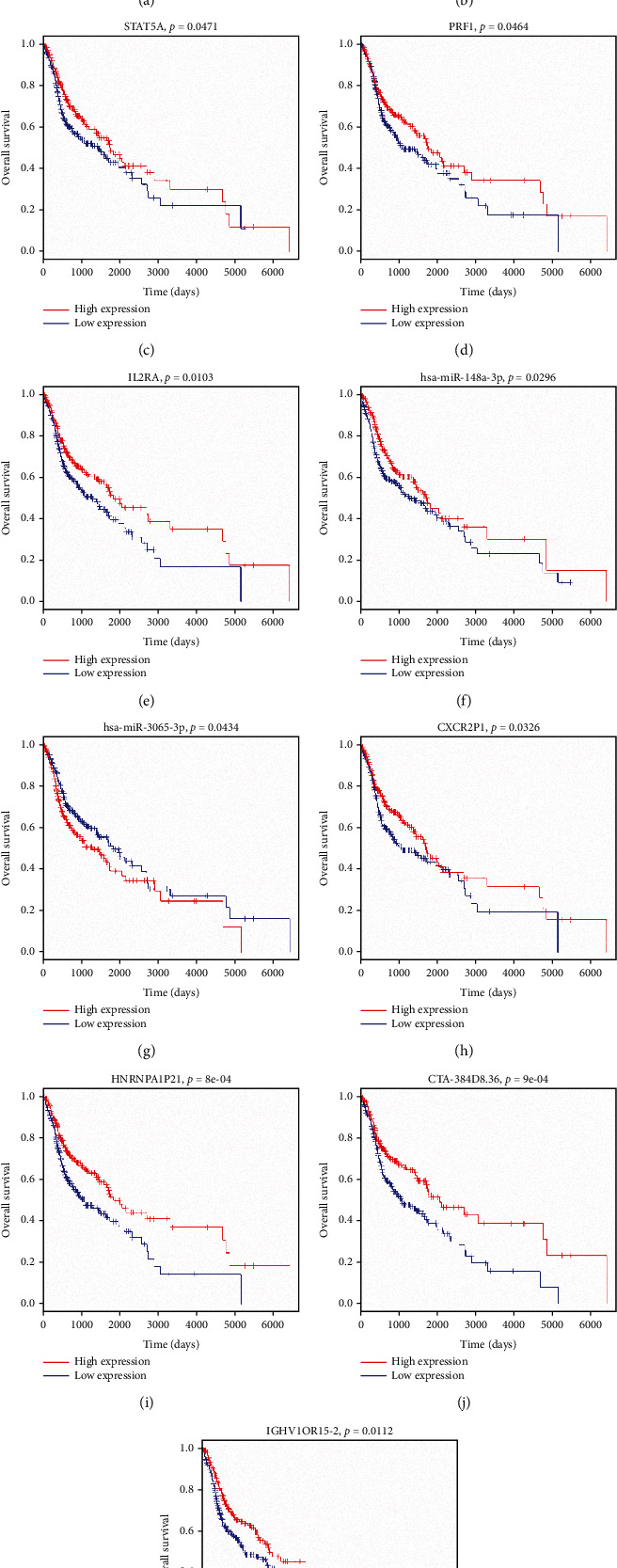
Kaplan-Meier survival curves of DEGs, DELs, and DEMs involved in the ceRNA network.

The PPI network constructed with the 97 DEGs in the ceRNA network contained 69 nodes and 203 edges. Twelve genes (FOXP3, IL10RA, CD274, CXCL9, IRF1, STAT5A, CXCL12, PRF1, IL2RA, MMP9, CSF1, and PTGS2) were prominent for having many connections with other genes (Figure 3(b)). Furthermore, survival analysis of DEGs, DELs, and DEMs involved in the ceRNA network was performed. The survival curves of five DEGs (FOXP3, IL10RA, STAT5A, PRF1, and IL2RA), two DEMs (miR-148a-3p and miR-3065-3p), and four DELs (CXCR2P1, HNRNPA1P21, CTA-384D8.36, and IGHV1OR15-2) are exhibited in Figures 4(a)–4(k). High expression levels of FOXP3, IL10RA, STAT5A, PRF1, IL2RA, miR-148a-3p, CXCR2P1, HNRNPA1P21, CTA-384D8.36, and IGHV1OR15-2 and low expression levels of miR-3065-3p were associated with longer OS in HNSCC patients (Figure 4).

### 3.7. Validation Using One Additional Independent Cohort

**Figure 5:**
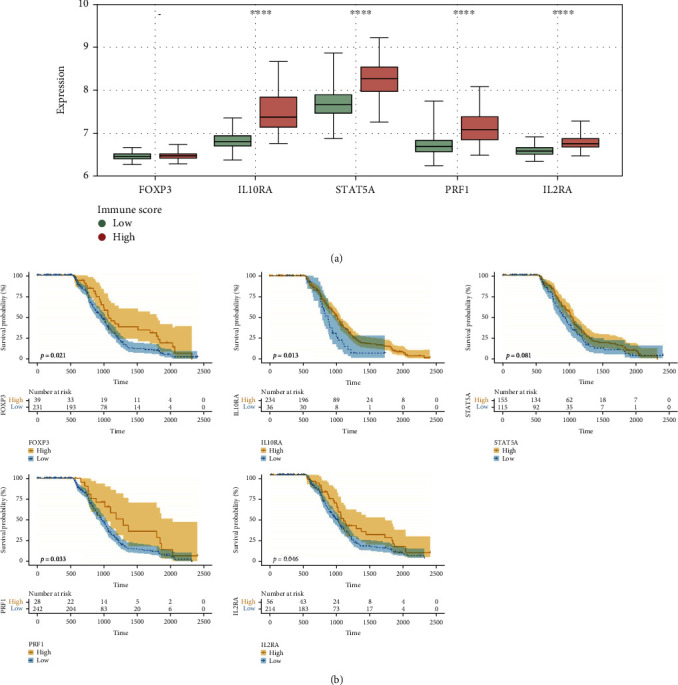
Verification of the different expression and survival analyses in GEO database. (a) Different expression levels of FOXP3, IL10RA, STAT5A, PRF1, and IL2RA in high and low immune score groups. (b) Kaplan-Meier survival curves of FOXP3, IL10RA, STAT5A, PRF1, and IL2RA in HNSCC patients.

To verify whether the eleven prognostic biomarkers above were differentially expressed and of prognostic significance in another independent HNSCC cohort, we downloaded GSE65858 from the GEO database for validation. However, for lacking sufficient RNA sequencing data of miRNAs and lncRNAs, we only successfully performed the differential expression and survival analysis of the five DEGs (FOXP3, IL10RA, STAT5A, PRF1, and IL2RA) between high and low immune score groups. As shown in Figure 5, IL10RA, PRF1, and IL2RA were not only significantly upregulated in patients high immune scores, but also their high expressions were associated with longer survival time, which were consistent with the results in the TCGA cohort.

### 3.8. Analysis of the Clinical Relevance and Immune Cell Infiltration for IL10RA, PRF1, and IL2RA

**Figure 6:**
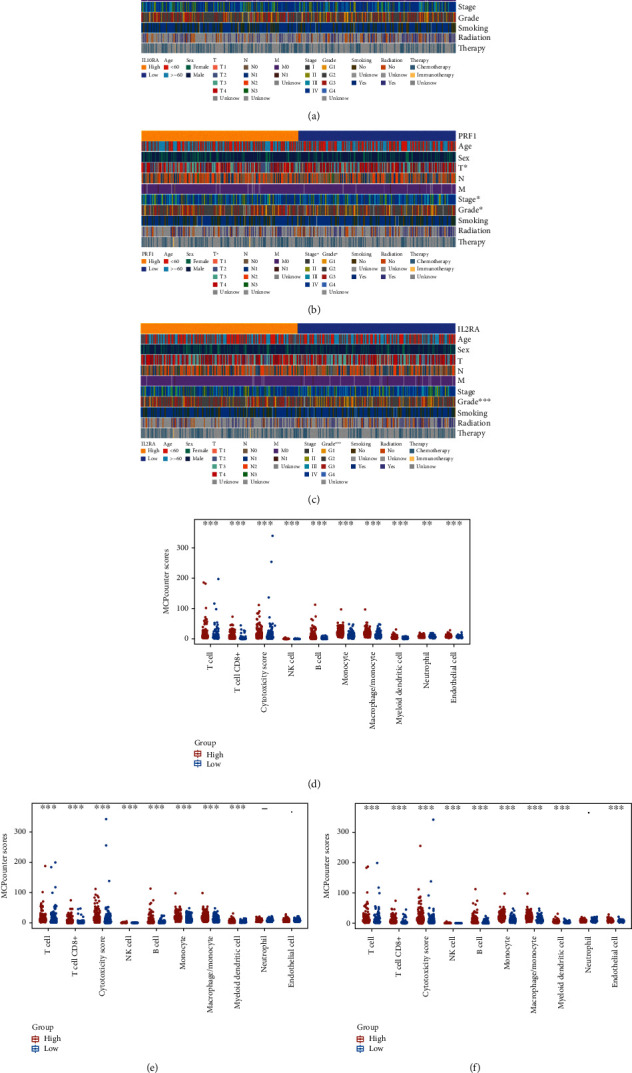
Analysis of the clinical characteristics and immune cell infiltration for IL10RA, PRF1, and IL2RA. The heatmaps of clinical characteristics in different expression groups of (a) IL10RA, (b) PRF1, and (c) IL2RA. The differences of immune cells in distinct expression groups of (d) IL10RA, (e) PRF1, and (f) IL2RA.

The distribution of clinical variables with corresponding expression subgroups was visualized (Figures 6(a)–6(c)). The results showed that the composition of T status, tumor stage, and clinical grade were significantly distinct between different PRF1 expression groups. And for the clinical relevance of IL2RA expression patterns, there was of significant difference in clinical grade, indicating that PRF1 and IL2RA might be involved in the mechanism of tumor progress. Unfortunately, we noticed no significant difference in clinical components between different IL10RA expression groups.

In addition, the immune infiltrating analysis showed that patients with high IL10RA, PRF1, and IL2RA expression exhibited high immune cells infiltration, such as CD8+ T cell, cytotoxic lymphocytes, NK cell, B cell, monocyte, and myeloid dendritic cell (Figures 6(d)–6(f)). This was also another evidence for that these three genes were elevated in patients high immune score groups.

## 4. Discussion

In this study, 468 HNSCC patients were divided into two groups based on immune/stromal scores using the ESTIMATE algorithm. ESTIMATE, a method that infers the fraction of immune and stromal cells in tumor samples based on gene expression [18], enables the quantification of the level of immune/stromal cells in TME in the form of a score. Survival analysis of the high- and low-score groups showed that patients with higher immune scores had relatively longer survival time, though the results showed no significant difference. Recent studies have asserted that infiltrating immune cells play crucial roles in tumor relapse, metastasis, therapy and prognosis [19–22]. And immune cell infiltration in HNSCC has been revealed to be involved in m^6^A methylation, alternative splicing, increased tumor mutation burden, and prognosis [23–25]. Therefore, a deeper understanding of immune cells and tumors at the molecular level is urgent.

The ceRNA network composed of 97 DEGs, 79 DELs, and 22 DEMs were constructed, and hsa-miR-149-5p, has-miR-17-5p, hsa-miR-3065-3p, hsa-miR-767-5p, and hsa-miR-96-5p were the top 5 nodes. Then, the PPI network identified 12 hub genes (FOXP3, IL10RA, CD274, CXCL9, IRF1, STAT5A, CXCL12, PRF1, IL2RA, MMP9, CSF1, and PTGS2), which might play important roles in the network. Among these genes, only PTGS2 was downregulated in the high immune score patients. KEGG analysis showed that PTGS2 was significantly involved in the terms “arachidonic acid metabolism,” “metabolic pathway,” and “chemical carcinogenesis.” PTGS2, also known as COX-2, is an enzyme critical for PGE2 that is associated with the enhancement of cancer cell survival, growth, migration, and invasion [26, 27]. PTGS2 is also associated with prognosis in multiple cancers [28]. In addition, for some tumors, tumor-derived PTGS2 serves an essential role in tumor immune evasion by inducing PGE2 to successfully evade elimination induced by type I interferon and/or T cells [26]. In the ceRNA network, PTGS2 was regulated by miR-148a-3p, and high expression of miR-148a-3p was significantly associated with increased OS in HNSCC patients (*P* = 0.0296; Figure 4). Accordingly, miR-148a-3p has been identified as a tumor suppressor in colorectal cancer, and its downregulation is associated with immune suppression [29]. The roles of miR-148a-3p/PTGS2 in the TME remain to be further investigated.

Of the upregulated hub genes in the PPI network, increased levels of IL10RA, PRF1, and IL2RA were significantly associated with longer survival time of HNSCC patients in both TCGA and GEO database. GO and KEGG analyses showed that IL2RA was significantly associated with “cytokine-cytokine receptor interaction” pathway. IL-2 interacts with IL2RA, which stimulates Tregs to express the transcription factors STAT5 and Foxp3, which play an essential role in Treg development and homeostasis [30, 31]. Tregs in the TME are plastic, endowing them with dual functionality [32]. Tregs are negatively correlated with OS in a majority of tumors [33]. However, they appear to be associated with improved OS in head and neck cancers [34], which is consistent with our finding that high expression levels of IL2RA were associated with favorable survival in HNSCC patients. IL10 is an essential regulator in immune homeostasis and notably serves this role through binding to its cell surface receptor, IL10RA [35]. GO and KEGG analyses showed that IL10RA was significantly associated with “cytokine-mediated signaling pathway,” “cytokine-cytokine receptor interaction,” and “Jak-STAT signaling pathway.” Song et al. suggested that high expression of IL10RA in HNSCC had better prognostic value, which is consistent with our findings [36]. The expression of PRF1 has been used to assess tumor-infiltrating lymphocytes in the tumor microenvironment and was related to the response of patients treated with anti-CTLA-4 therapy and anti-PD-1/PD-L1 therapy [37, 38]. Furthermore, Yang et al. asserted that a high expression level of PRF1 provided an appropriate microenvironment for anti-CTLA-4 and anti-PD-1/PD-L1 therapy in type I and II ovarian cancer [39]. Similarly, in this study, overexpression of PRF1 in HNSCC patients with a high immune score was identified, and its overexpression was associated with increased OS. The above findings, combined with the results of the clinical relevance and immune cell infiltration for IL10RA, PRF1, and IL2RA, emphasized that PRF1 and IL2RA might be involved in the mechanism of tumor progress and also provided evidence for the overexpression of the three genes in immune score groups.

Interestingly, in the ceRNA network, miR-3065-3p and the lncRNAs CXCR2P1, HNRNPA1P21, CTA-384D8.36, and IGHV1OR15-2 were significantly correlated with OS in HNSCC patients in the TCGA database; the relevant interactions within the network include the following: miR-3065-3p/IL2RA; CXCR2P1/miR-210-3p/FOXP3; HNRNPA1P21/miR-767-5p/IL10RA; CTA-384D8.36/miR-149-5p/STAT5A; CTA-384D8.36/miR-149-5p/PRF1; and IGHV1OR15-2/miR-744-3p/IL2RA. MiR-3065-3p was downregulated in high immune score patients, and patients with low miR-3065-3p expression had improved overall survival. The overexpression of the lncRNAs CXCR2P1, HNRNPA1P21, CTA-384D8.36, and IGHV1OR15-2 were correlated with longer overall survival for HNSCC patients. Among these, CXCR2P1 has been speculated to be related to immune checkpoints PD-1, PD-L1, and CTLA4, which are crucial for successful cancer immunotherapy that contribute to the immune response [40, 41], but there is still a lack of direct evidence. Herein, the CXCR2P1/miR-210-3p/FOXP3 axis identified in the ceRNA network may provide clues for future studies of the biological functions of CXCR2P1 in the TME of HNSCC. For HNRNPA1P21, CTA-384D8.36, and IGHV1OR15-2, their prognostic roles were first reported in HNSCC. Although these prognosis-related miRNAs and lncRNAs could not be verified due to the lack of RNA sequencing and survival data in GEO database, their biological functions in the TME of HNSCC should not be ignored, and they should be further studied when enough clinical samples and information were provided. In addition, another limitation of the present study should also be taken into consideration. We inferred the level of infiltrating stromal and immune cells in tumor tissues by using ESTIMATE algorithm and not by direct analysis of actual infiltrating cells, which may provide relevant information but is not certainly related to the actual cell content of the TME.

In conclusion, we estimated the level of infiltrating stromal and immune cells in the TME of HNSCC patients and constructed a ceRNA network, in which FOXP3, IL10RA, STAT5A, PRF1, IL2RA, miR-148a-3p, miR-3065-3p, CXCR2P1, HNRNPA1P21, CTA-384D8.36, and IGHV1OR15-2 were significantly correlated with the OS of HNSCC patients. Besides, the expression and survival roles of IL10RA, PRF1, and IL2RA were verified in another GEO cohort. This might provide novel targets in the TME of HNSCC and contribute to therapeutic intervention and prognosis evaluation.

## Data Availability

Datasets used in this study were obtained from open public databases (https://tcga-data.nci.nih.gov/tcga/, https://www.ncbi.nlm.nih.gov/geo/), but the datasets analyzed in this study are available from the corresponding authors on request.

## Supplementary Materials

Supplementary 1 Table S1. Clinical characteristic of HNSCC patients.

Supplementary 2 Table S2. Immune scores and stromal scores of HNSCC patients.
