# Supplementary material for: Immune Infiltration-Related ceRNA Network Revealing Potential Biomarkers for Prognosis of Head and Neck Squamous Cell Carcinoma
Source: Dis Markers. 2022 Sep 2;2022:1014347. doi: 10.1155/2022/1014347 (PMC9463596; doi:10.1155/2022/1014347)
Supplement: Supplementary 1 — Table S1. Clinical characteristic of HNSCC patients. [file 1014347.f1.pdf]

Table S2. Immune scores and stromal scores of HNSCC patients.

Sample_title	ImmuneScore	StromalScore	IS_group	SS_group
TCGA-4P-AA8J-01	744.9118096	-24.60699006	-IS_low	-SS_high
TCGA-BA-4074-01	2387.052646	1540.9361	-IS_high	-SS_high
TCGA-BA-4076-01	219.6101738	-306.5797652	-IS_low	-SS_high
TCGA-BA-4078-01	913.6892665	-977.7040807	-IS_high	-SS_low
TCGA-BA-5151-01	54.24710438	118.5839469	-IS_low	-SS_high
TCGA-BA-5152-01	789.5218531	-94.20821946	-IS_low	-SS_high
TCGA-BA-5153-01	722.8795049	-326.2851405	-IS_low	-SS_low
TCGA-BA-5555-01	1423.953214	-49.76812733	-IS_high	-SS_high
TCGA-BA-5556-01	-136.7746337	-997.0456213	-IS_low	-SS_low
TCGA-BA-5558-01	45.69790162	772.9578421	-IS_low	-SS_high
TCGA-BA-6868-01	303.9798985	631.6020056	-IS_low	-SS_high
TCGA-BA-6869-01	2207.949626	-168.5787161	-IS_high	-SS_high
TCGA-BA-6870-01	-8.492410359	-1311.462322	-IS_low	-SS_low
TCGA-BA-6872-01	563.7456843	81.76975275	-IS_low	-SS_high
TCGA-BA-6873-01	-632.4789314	-1201.970758	-IS_low	-SS_low
TCGA-BA-7269-01	1900.47078	864.4764062	-IS_high	-SS_high
TCGA-BA-A4IF-01	309.481085	-1319.94043	-IS_low	-SS_low
TCGA-BA-A4IH-01	1968.101986	-31.67107765	-IS_high	-SS_high
TCGA-BA-A6D8-01	483.7147923	-60.15055796	-IS_low	-SS_high
TCGA-BA-A6DA-01	2167.184398	398.3446259	-IS_high	-SS_high
TCGA-BA-A6DB-01	-165.2253223	-1841.251049	-IS_low	-SS_low
TCGA-BA-A6DD-01	973.4608963	389.8303988	-IS_high	-SS_high
TCGA-BA-A6DE-01	-16.17605189	-923.9518268	-IS_low	-SS_low
TCGA-BA-A6DG-01	-277.1008989	-1430.022612	-IS_low	-SS_low
TCGA-BA-A6DI-01	745.2437512	-125.6409253	-IS_low	-SS_high
TCGA-BA-A6DJ-01	509.8911605	-1234.296429	-IS_low	-SS_low
TCGA-BA-A6DL-01	-445.5151318	-1917.586777	-IS_low	-SS_low
TCGA-BA-A8YP-01	1803.74201	602.638002	-IS_high	-SS_high
TCGA-BB-4217-01	1173.8435	42.9840528	-IS_high	-SS_high
TCGA-BB-4223-01	427.346669	-608.0295551	-IS_low	-SS_low
TCGA-BB-4224-01	2375.47192	1213.533626	-IS_high	-SS_high
TCGA-BB-4225-01	1906.748399	304.0557585	-IS_high	-SS_high
TCGA-BB-4227-01	323.4847228	-420.1796974	-IS_low	-SS_low
TCGA-BB-4228-01	-114.8951257	-966.2547063	-IS_low	-SS_low
TCGA-BB-8596-01	880.4821528	158.0847736	-IS_high	-SS_high
TCGA-BB-8601-01	2209.091918	-676.0665343	-IS_high	-SS_low
TCGA-BB-A5HY-01	502.3910241	255.3527596	-IS_low	-SS_high
TCGA-BB-A5HZ-01	1163.549786	-720.0439208	-IS_high	-SS_low
TCGA-BB-A6UO-01	40.00965397	-1200.211517	-IS_low	-SS_low
TCGA-C9-A47Z-01	984.4363696	-602.9953427	-IS_high	-SS_low
TCGA-C9-A480-01	-324.3719876	-1573.210957	-IS_low	-SS_low
TCGA-CN-4722-01	794.008451	-244.7600739	-IS_low	-SS_high

TCGA-CN-4723-01	549.2002444	-506.7237226	-IS_low	-SS_low
TCGA-CN-4725-01	361.2106576	-1362.502784	-IS_low	-SS_low
TCGA-CN-4726-01	-142.0527279	-522.6364674	-IS_low	-SS_low
TCGA-CN-4728-01	-172.2499406	-1026.86749	-IS_low	-SS_low
TCGA-CN-4729-01	714.1543597	214.4229802	-IS_low	-SS_high
TCGA-CN-4730-01	690.9668792	92.73881341	-IS_low	-SS_high
TCGA-CN-4731-01	1685.730012	1229.830536	-IS_high	-SS_high
TCGA-CN-4733-01	1628.729516	-404.3176755	-IS_high	-SS_low
TCGA-CN-4735-01	1385.867934	150.9830225	-IS_high	-SS_high
TCGA-CN-4736-01	677.8265781	-89.96328481	-IS_low	-SS_high
TCGA-CN-4737-01	1991.166067	1503.96036	-IS_high	-SS_high
TCGA-CN-4738-01	2381.99338	-40.00623828	-IS_high	-SS_high
TCGA-CN-4739-01	1109.488364	-494.3671943	-IS_high	-SS_low
TCGA-CN-4740-01	745.0531287	-810.3357888	-IS_low	-SS_low
TCGA-CN-4741-01	108.1719107	-1164.594043	-IS_low	-SS_low
TCGA-CN-4742-01	429.7420478	-518.3149048	-IS_low	-SS_low
TCGA-CN-5355-01	441.5982616	357.3822048	-IS_low	-SS_high
TCGA-CN-5356-01	89.41347631	-529.2140499	-IS_low	-SS_low
TCGA-CN-5358-01	936.2783089	-619.1548355	-IS_high	-SS_low
TCGA-CN-5359-01	2386.876639	1083.970263	-IS_high	-SS_high
TCGA-CN-5360-01	-621.3320327	-411.1202633	-IS_low	-SS_low
TCGA-CN-5361-01	180.7657201	-1404.9557	-IS_low	-SS_low
TCGA-CN-5363-01	1076.826634	985.1463231	-IS_high	-SS_high
TCGA-CN-5364-01	474.6159646	-960.9649297	-IS_low	-SS_low
TCGA-CN-5365-01	1126.655029	1154.873098	-IS_high	-SS_high
TCGA-CN-5366-01	725.0364963	-937.1518351	-IS_low	-SS_low
TCGA-CN-5367-01	1048.104826	-686.3250821	-IS_high	-SS_low
TCGA-CN-5369-01	1971.12212	1624.026865	-IS_high	-SS_high
TCGA-CN-5370-01	1088.097386	1471.200956	-IS_high	-SS_high
TCGA-CN-5373-01	1157.356139	-137.9358259	-IS_high	-SS_high
TCGA-CN-5374-01	1896.611628	-818.4205649	-IS_high	-SS_low
TCGA-CN-6010-01	-117.7941699	-499.6049782	-IS_low	-SS_low
TCGA-CN-6011-01	468.3922499	-282.3539619	-IS_low	-SS_high
TCGA-CN-6012-01	1827.068059	-817.5842497	-IS_high	-SS_low
TCGA-CN-6013-01	421.784925	75.99868009	-IS_low	-SS_high
TCGA-CN-6016-01	932.5001487	-669.3720578	-IS_high	-SS_low
TCGA-CN-6017-01	-295.3917505	-958.4419988	-IS_low	-SS_low
TCGA-CN-6018-01	29.72019811	-197.7598744	-IS_low	-SS_high
TCGA-CN-6019-01	-57.00281378	-1335.15406	-IS_low	-SS_low
TCGA-CN-6020-01	1067.131987	-215.9360876	-IS_high	-SS_high
TCGA-CN-6021-01	1686.240589	-304.914487	-IS_high	-SS_high
TCGA-CN-6022-01	2496.847106	1438.230113	-IS_high	-SS_high
TCGA-CN-6023-01	1252.513904	273.7742764	-IS_high	-SS_high
TCGA-CN-6024-01	358.6848007	28.97575203	-IS_low	-SS_high

TCGA-CN-6988-01	-170.6987239	523.3840682	-IS_low	-SS_high
TCGA-CN-6989-01	2033.287955	883.2277751	-IS_high	-SS_high
TCGA-CN-6994-01	516.5376539	-252.4993084	-IS_low	-SS_high
TCGA-CN-6995-01	777.0064926	-451.5949964	-IS_low	-SS_low
TCGA-CN-6996-01	796.8891079	-322.5847111	-IS_low	-SS_low
TCGA-CN-6998-01	862.0024098	-917.7971052	-IS_low	-SS_low
TCGA-CN-A497-01	66.03861171	-1761.08467	-IS_low	-SS_low
TCGA-CN-A498-01	796.958788	-1233.176273	-IS_low	-SS_low
TCGA-CN-A499-01	686.8345599	-1000.5279	-IS_low	-SS_low
TCGA-CN-A49A-01	1874.052421	-41.43169641	-IS_high	-SS_high
TCGA-CN-A49B-01	1797.880036	220.1696919	-IS_high	-SS_high
TCGA-CN-A49C-01	1339.646398	555.951197	-IS_high	-SS_high
TCGA-CN-A63T-01	93.43189665	-1510.066528	-IS_low	-SS_low
TCGA-CN-A63U-01	1657.833967	-632.1689796	-IS_high	-SS_low
TCGA-CN-A63V-01	814.0945183	-464.1896294	-IS_low	-SS_low
TCGA-CN-A63W-01	538.9582123	321.0012018	-IS_low	-SS_high
TCGA-CN-A641-01	1185.667689	-279.4608219	-IS_high	-SS_high
TCGA-CN-A642-01	1941.630758	-104.2687392	-IS_high	-SS_high
TCGA-CN-A6UY-01	1410.128698	-947.8948364	-IS_high	-SS_low
TCGA-CN-A6V6-01	2801.345211	1989.277545	-IS_high	-SS_high
TCGA-CN-A6V7-01	2102.51843	297.4724907	-IS_high	-SS_high
TCGA-CQ-5324-01	158.022892	-1029.98967	-IS_low	-SS_low
TCGA-CQ-5325-01	393.792842	-1790.78973	-IS_low	-SS_low
TCGA-CQ-5326-01	-171.4539323	-864.515702	-IS_low	-SS_low
TCGA-CQ-5327-01	-416.0391445	-1021.251179	-IS_low	-SS_low
TCGA-CQ-5329-01	-438.2900623	-2062.808319	-IS_low	-SS_low
TCGA-CQ-5330-01	358.4491134	-441.2238908	-IS_low	-SS_low
TCGA-CQ-5331-01	1941.90025	1582.12723	-IS_high	-SS_high
TCGA-CQ-5332-01	2157.908622	-645.889057	-IS_high	-SS_low
TCGA-CQ-5333-01	1808.314498	773.7767762	-IS_high	-SS_high
TCGA-CQ-5334-01	782.0423415	426.8951366	-IS_low	-SS_high
TCGA-CQ-6218-01	508.8822484	-624.1041301	-IS_low	-SS_low
TCGA-CQ-6219-01	1101.625373	9.592691395	-IS_high	-SS_high
TCGA-CQ-6220-01	2389.197087	798.297356	-IS_high	-SS_high
TCGA-CQ-6222-01	-383.2934947	-707.738409	-IS_low	-SS_low
TCGA-CQ-6223-01	-252.1441184	-520.2847169	-IS_low	-SS_low
TCGA-CQ-6224-01	1424.745613	-58.02638354	-IS_high	-SS_high
TCGA-CQ-6225-01	256.9147021	-524.3459632	-IS_low	-SS_low
TCGA-CQ-6227-01	119.9334457	-1269.288956	-IS_low	-SS_low
TCGA-CQ-6228-01	2735.002705	1226.223236	-IS_high	-SS_high
TCGA-CQ-6229-01	1518.280567	-202.1759217	-IS_high	-SS_high
TCGA-CQ-7065-01	247.4966869	-1633.396862	-IS_low	-SS_low
TCGA-CQ-7069-01	-114.9031524	-751.2147056	-IS_low	-SS_low
TCGA-CQ-7071-01	-114.032807	-1092.142088	-IS_low	-SS_low

TCGA-CQ-7072-01	780.0197917	-171.529473	-IS_low	-SS_high
TCGA-CQ-A4C6-01	1747.49596	-957.172975	-IS_high	-SS_low
TCGA-CQ-A4C9-01	639.8389824	491.2561138	-IS_low	-SS_high
TCGA-CQ-A4CA-01	263.5477352	-2092.213103	-IS_low	-SS_low
TCGA-CQ-A4CB-01	-620.6941585	-195.4192986	-IS_low	-SS_high
TCGA-CQ-A4CD-01	483.9330636	-1093.767719	-IS_low	-SS_low
TCGA-CQ-A4CE-01	-1088.391009	-1959.356159	-IS_low	-SS_low
TCGA-CQ-A4CG-01	2177.383549	763.8700779	-IS_high	-SS_high
TCGA-CQ-A4CH-01	-631.8872883	-1543.567025	-IS_low	-SS_low
TCGA-CQ-A4CI-01	1343.934296	-1280.165934	-IS_high	-SS_low
TCGA-CR-5243-01	687.8406979	728.4858014	-IS_low	-SS_high
TCGA-CR-5247-01	1389.221973	30.26028375	-IS_high	-SS_high
TCGA-CR-5248-01	286.0185012	-932.0340517	-IS_low	-SS_low
TCGA-CR-5249-01	592.6283382	-410.5703712	-IS_low	-SS_low
TCGA-CR-5250-01	179.8651411	-1128.322418	-IS_low	-SS_low
TCGA-CR-6467-01	669.9783811	-893.0934135	-IS_low	-SS_low
TCGA-CR-6470-01	212.2759522	241.6176371	-IS_low	-SS_high
TCGA-CR-6471-01	253.0885617	-259.6162283	-IS_low	-SS_high
TCGA-CR-6473-01	960.12555	-1017.674769	-IS_high	-SS_low
TCGA-CR-6474-01	2077.039583	929.0174918	-IS_high	-SS_high
TCGA-CR-6477-01	-150.4200056	-85.53656467	-IS_low	-SS_high
TCGA-CR-6480-01	2801.351255	678.8441023	-IS_high	-SS_high
TCGA-CR-6481-01	1405.863134	741.4797711	-IS_high	-SS_high
TCGA-CR-6482-01	1633.837006	143.4543126	-IS_high	-SS_high
TCGA-CR-6484-01	1294.082148	-1649.970354	-IS_high	-SS_low
TCGA-CR-6487-01	1209.101583	-430.3161627	-IS_high	-SS_low
TCGA-CR-6488-01	403.6400947	-230.3038183	-IS_low	-SS_high
TCGA-CR-6491-01	2020.171998	-914.8453979	-IS_high	-SS_low
TCGA-CR-6492-01	1606.521159	220.6440941	-IS_high	-SS_high
TCGA-CR-6493-01	273.9487853	156.2720119	-IS_low	-SS_high
TCGA-CR-7364-01	1532.374212	-421.9013814	-IS_high	-SS_low
TCGA-CR-7365-01	1624.935429	-798.9449158	-IS_high	-SS_low
TCGA-CR-7367-01	1775.923629	-1178.433569	-IS_high	-SS_low
TCGA-CR-7368-01	409.6609342	-640.4401745	-IS_low	-SS_low
TCGA-CR-7369-01	1114.446652	-658.259197	-IS_high	-SS_low
TCGA-CR-7370-01	-423.8389109	-1624.009422	-IS_low	-SS_low
TCGA-CR-7371-01	1437.939677	-1085.784864	-IS_high	-SS_low
TCGA-CR-7372-01	1778.685231	1670.553176	-IS_high	-SS_high
TCGA-CR-7373-01	1320.156269	1068.549102	-IS_high	-SS_high
TCGA-CR-7374-01	779.8723218	-688.6911207	-IS_low	-SS_low
TCGA-CR-7376-01	1259.257679	456.6499458	-IS_high	-SS_high
TCGA-CR-7377-01	-161.7963581	-1291.441473	-IS_low	-SS_low
TCGA-CR-7379-01	111.9162778	-1492.619046	-IS_low	-SS_low
TCGA-CR-7380-01	611.3956034	-1478.57981	-IS_low	-SS_low

TCGA-CR-7382-01	2529.555589	831.4513266	-IS_high	-SS_high
TCGA-CR-7383-01	821.0677558	-528.5441948	-IS_low	-SS_low
TCGA-CR-7385-01	610.804118	-20.34581245	-IS_low	-SS_high
TCGA-CR-7386-01	2259.722168	-722.2235625	-IS_high	-SS_low
TCGA-CR-7388-01	996.5670256	122.640974	-IS_high	-SS_high
TCGA-CR-7389-01	1048.448193	-76.83607404	-IS_high	-SS_high
TCGA-CR-7390-01	387.5731974	-1075.917039	-IS_low	-SS_low
TCGA-CR-7391-01	282.6589298	-1053.329005	-IS_low	-SS_low
TCGA-CR-7392-01	429.6338542	-745.7500832	-IS_low	-SS_low
TCGA-CR-7393-01	-298.3127103	-1462.428676	-IS_low	-SS_low
TCGA-CR-7394-01	1218.992911	301.6017574	-IS_high	-SS_high
TCGA-CR-7395-01	1106.34564	-219.4669129	-IS_high	-SS_high
TCGA-CR-7397-01	1321.839239	221.2112468	-IS_high	-SS_high
TCGA-CR-7398-01	1983.636398	731.7535493	-IS_high	-SS_high
TCGA-CR-7399-01	245.6979961	-1184.698719	-IS_low	-SS_low
TCGA-CR-7401-01	27.16971244	-253.3755352	-IS_low	-SS_high
TCGA-CR-7402-01	1276.279379	853.6893861	-IS_high	-SS_high
TCGA-CR-7404-01	845.6755349	505.871106	-IS_low	-SS_high
TCGA-CV-5430-01	702.2112273	-1023.056232	-IS_low	-SS_low
TCGA-CV-5431-01	2287.771334	-416.8117299	-IS_high	-SS_low
TCGA-CV-5432-01	2090.334055	-281.1949895	-IS_high	-SS_high
TCGA-CV-5434-01	955.7720672	369.1186707	-IS_high	-SS_high
TCGA-CV-5435-01	779.406668	-728.8059869	-IS_low	-SS_low
TCGA-CV-5436-01	923.670753	-1099.119366	-IS_high	-SS_low
TCGA-CV-5439-01	1122.321125	340.1679281	-IS_high	-SS_high
TCGA-CV-5440-01	2150.004258	1253.350164	-IS_high	-SS_high
TCGA-CV-5441-01	1695.46972	-519.5396802	-IS_high	-SS_low
TCGA-CV-5442-01	1846.333696	-461.344913	-IS_high	-SS_low
TCGA-CV-5443-01	1388.433774	-811.9160276	-IS_high	-SS_low
TCGA-CV-5444-01	115.5867277	-1190.244995	-IS_low	-SS_low
TCGA-CV-5966-01	379.3475758	299.1745637	-IS_low	-SS_high
TCGA-CV-5970-01	2774.799962	258.4175908	-IS_high	-SS_high
TCGA-CV-5971-01	1383.693156	-148.4458143	-IS_high	-SS_high
TCGA-CV-5973-01	158.2001075	185.801649	-IS_low	-SS_high
TCGA-CV-5976-01	2490.589004	325.1093585	-IS_high	-SS_high
TCGA-CV-5977-01	1421.453849	-480.9277893	-IS_high	-SS_low
TCGA-CV-5978-01	-319.4339771	-1204.357025	-IS_low	-SS_low
TCGA-CV-5979-01	694.8113764	-695.7518983	-IS_low	-SS_low
TCGA-CV-6003-01	1846.311795	-1419.538995	-IS_high	-SS_low
TCGA-CV-6433-01	-103.2290733	-1293.3882	-IS_low	-SS_low
TCGA-CV-6436-01	-473.8814658	-1785.713498	-IS_low	-SS_low
TCGA-CV-6441-01	993.2547468	-1088.948056	-IS_high	-SS_low
TCGA-CV-6933-01	2620.140157	1762.468421	-IS_high	-SS_high
TCGA-CV-6934-01	1164.657499	-273.719291	-IS_high	-SS_high

TCGA-CV-6935-01	-338.8392735	-1376.647553	-IS_low	-SS_low
TCGA-CV-6936-01	481.4671113	-360.3138425	-IS_low	-SS_low
TCGA-CV-6937-01	391.5369391	646.9698363	-IS_low	-SS_high
TCGA-CV-6938-01	1742.479888	302.658903	-IS_high	-SS_high
TCGA-CV-6939-01	569.2153467	-673.5232336	-IS_low	-SS_low
TCGA-CV-6940-01	2123.513093	-167.1044566	-IS_high	-SS_high
TCGA-CV-6941-01	621.6700364	-287.0350701	-IS_low	-SS_high
TCGA-CV-6942-01	375.7592319	-1327.902359	-IS_low	-SS_low
TCGA-CV-6943-01	362.8227273	-357.1239401	-IS_low	-SS_low
TCGA-CV-6945-01	1821.886844	-532.7558216	-IS_high	-SS_low
TCGA-CV-6948-01	150.6130039	-1090.879815	-IS_low	-SS_low
TCGA-CV-6950-01	1970.399469	1017.630338	-IS_high	-SS_high
TCGA-CV-6951-01	1314.982982	-720.6655841	-IS_high	-SS_low
TCGA-CV-6952-01	-399.6581252	-1109.934351	-IS_low	-SS_low
TCGA-CV-6953-01	875.1673054	-232.1431788	-IS_low	-SS_high
TCGA-CV-6954-01	258.4583505	-533.3270347	-IS_low	-SS_low
TCGA-CV-6955-01	2718.754266	-312.692186	-IS_high	-SS_low
TCGA-CV-6956-01	1982.858916	-489.4905029	-IS_high	-SS_low
TCGA-CV-6959-01	167.8692856	-1537.23997	-IS_low	-SS_low
TCGA-CV-6960-01	220.5694184	-569.2703462	-IS_low	-SS_low
TCGA-CV-6961-01	1433.094756	-357.5047595	-IS_high	-SS_low
TCGA-CV-6962-01	163.8901779	-1151.664494	-IS_low	-SS_low
TCGA-CV-7089-01	1127.941354	-384.8814293	-IS_high	-SS_low
TCGA-CV-7090-01	129.4925441	-290.3892766	-IS_low	-SS_high
TCGA-CV-7091-01	724.8363103	369.0007275	-IS_low	-SS_high
TCGA-CV-7095-01	966.4484134	-534.5284293	-IS_high	-SS_low
TCGA-CV-7097-01	-275.2337066	-922.8565798	-IS_low	-SS_low
TCGA-CV-7099-01	2013.165937	342.9659784	-IS_high	-SS_high
TCGA-CV-7100-01	108.3440559	-1147.8195	-IS_low	-SS_low
TCGA-CV-7101-01	-246.2196538	-891.0429018	-IS_low	-SS_low
TCGA-CV-7102-01	149.4459504	-993.8968929	-IS_low	-SS_low
TCGA-CV-7103-01	2115.839987	722.727134	-IS_high	-SS_high
TCGA-CV-7104-01	1137.749821	-434.2897939	-IS_high	-SS_low
TCGA-CV-7177-01	1574.954631	-742.3246103	-IS_high	-SS_low
TCGA-CV-7178-01	102.3756655	-608.7900429	-IS_low	-SS_low
TCGA-CV-7180-01	2077.699084	44.16605074	-IS_high	-SS_high
TCGA-CV-7183-01	-510.6785758	-833.4245679	-IS_low	-SS_low
TCGA-CV-7235-01	451.0211225	21.67594789	-IS_low	-SS_high
TCGA-CV-7236-01	1629.913941	-251.8025403	-IS_high	-SS_high
TCGA-CV-7238-01	2769.279532	-412.9740928	-IS_high	-SS_low
TCGA-CV-7242-01	1199.183825	-839.2206295	-IS_high	-SS_low
TCGA-CV-7245-01	1358.06419	-192.8045636	-IS_high	-SS_high
TCGA-CV-7247-01	444.7107743	516.9540937	-IS_low	-SS_high
TCGA-CV-7248-01	1904.133055	-317.3539511	-IS_high	-SS_low

TCGA-CV-7250-01	1094.544678	1203.661209	-IS_high	-SS_high
TCGA-CV-7252-01	1072.900516	-148.2851199	-IS_high	-SS_high
TCGA-CV-7253-01	1222.816015	-280.5966191	-IS_high	-SS_high
TCGA-CV-7254-01	477.1263039	-1600.556696	-IS_low	-SS_low
TCGA-CV-7255-01	-640.0615435	-1163.099173	-IS_low	-SS_low
TCGA-CV-7261-01	1358.403684	394.4964495	-IS_high	-SS_high
TCGA-CV-7263-01	-352.6667295	-1305.69779	-IS_low	-SS_low
TCGA-CV-7406-01	473.6654638	-67.62305199	-IS_low	-SS_high
TCGA-CV-7407-01	2476.073069	-500.6388344	-IS_high	-SS_low
TCGA-CV-7410-01	1310.926045	-18.50200042	-IS_high	-SS_high
TCGA-CV-7411-01	-41.96844285	-472.771874	-IS_low	-SS_low
TCGA-CV-7413-01	758.8031088	-1180.555286	-IS_low	-SS_low
TCGA-CV-7414-01	373.6248051	-931.6249478	-IS_low	-SS_low
TCGA-CV-7415-01	99.37812275	-833.24427	-IS_low	-SS_low
TCGA-CV-7416-01	591.5357636	1270.624428	-IS_low	-SS_high
TCGA-CV-7418-01	855.8716815	-59.71308458	-IS_low	-SS_high
TCGA-CV-7421-01	2641.279921	-528.6437472	-IS_high	-SS_low
TCGA-CV-7422-01	1548.232493	981.3079972	-IS_high	-SS_high
TCGA-CV-7423-01	-784.4063006	-1274.008724	-IS_low	-SS_low
TCGA-CV-7424-01	118.9773674	129.3073724	-IS_low	-SS_high
TCGA-CV-7425-01	1352.500127	1026.301889	-IS_high	-SS_high
TCGA-CV-7427-01	2033.736331	1697.285139	-IS_high	-SS_high
TCGA-CV-7428-01	88.33776722	216.7742255	-IS_low	-SS_high
TCGA-CV-7429-01	1570.544956	-281.7901737	-IS_high	-SS_high
TCGA-CV-7430-01	522.3206541	-265.9229886	-IS_low	-SS_high
TCGA-CV-7432-01	1463.716682	-592.6193796	-IS_high	-SS_low
TCGA-CV-7433-01	816.799795	28.60222816	-IS_low	-SS_high
TCGA-CV-7434-01	880.5021267	-637.1743257	-IS_high	-SS_low
TCGA-CV-7435-01	1304.318538	746.1689877	-IS_high	-SS_high
TCGA-CV-7437-01	1127.61714	-80.05925645	-IS_high	-SS_high
TCGA-CV-7438-01	291.4271218	-767.5349831	-IS_low	-SS_low
TCGA-CV-7440-01	1658.219114	259.8360557	-IS_high	-SS_high
TCGA-CV-7446-01	1720.940888	585.1756168	-IS_high	-SS_high
TCGA-CV-7568-01	1745.703679	-98.57110866	-IS_high	-SS_high
TCGA-CV-A45O-01	607.5387981	-393.3456173	-IS_low	-SS_low
TCGA-CV-A45P-01	586.2921313	-98.36729463	-IS_low	-SS_high
TCGA-CV-A45Q-01	72.66241219	-729.6533143	-IS_low	-SS_low
TCGA-CV-A45R-01	2248.123177	896.7116869	-IS_high	-SS_high
TCGA-CV-A45T-01	433.6401223	-166.2192015	-IS_low	-SS_high
TCGA-CV-A45U-01	323.5902395	-487.7410987	-IS_low	-SS_low
TCGA-CV-A45V-01	1277.568648	-542.5923988	-IS_high	-SS_low
TCGA-CV-A45W-01	866.0948844	595.836163	-IS_low	-SS_high
TCGA-CV-A45X-01	1136.003631	-450.6748896	-IS_high	-SS_low
TCGA-CV-A45Y-01	1001.225687	308.0615324	-IS_high	-SS_high

TCGA-CV-A45Z-01	701.9719398	-339.6609978	-IS_low	-SS_low
TCGA-CV-A460-01	2714.207734	133.8356378	-IS_high	-SS_high
TCGA-CV-A461-01	1115.279774	389.4523307	-IS_high	-SS_high
TCGA-CV-A463-01	-712.0979058	-1309.628909	-IS_low	-SS_low
TCGA-CV-A464-01	1791.602314	-225.6026944	-IS_high	-SS_high
TCGA-CV-A465-01	374.6728561	23.94215236	-IS_low	-SS_high
TCGA-CV-A468-01	58.10777555	-1258.683171	-IS_low	-SS_low
TCGA-CV-A6JD-01	2753.547361	479.6679864	-IS_high	-SS_high
TCGA-CV-A6JE-01	1508.567571	587.6261681	-IS_high	-SS_high
TCGA-CV-A6JM-01	795.7836508	-696.6729626	-IS_low	-SS_low
TCGA-CV-A6JN-01	1901.83497	-524.4791099	-IS_high	-SS_low
TCGA-CV-A6JO-01	805.8222158	-78.8990372	-IS_low	-SS_high
TCGA-CV-A6JT-01	1063.577791	-563.8532724	-IS_high	-SS_low
TCGA-CV-A6JU-01	1175.378521	469.8179967	-IS_high	-SS_high
TCGA-CV-A6JY-01	1171.745032	141.4324075	-IS_high	-SS_high
TCGA-CV-A6JZ-01	251.0210956	-1142.584701	-IS_low	-SS_low
TCGA-CV-A6K0-01	2420.262089	-371.1034635	-IS_high	-SS_low
TCGA-CV-A6K1-01	2026.40667	551.807057	-IS_high	-SS_high
TCGA-CV-A6K2-01	998.9503992	22.75924856	-IS_high	-SS_high
TCGA-CX-7085-01	1199.418963	154.7678128	-IS_high	-SS_high
TCGA-CX-7086-01	777.2445194	-645.8437656	-IS_low	-SS_low
TCGA-CX-A4AQ-01	154.8262036	-391.3578131	-IS_low	-SS_low
TCGA-D6-6515-01	952.1936077	-557.2166867	-IS_high	-SS_low
TCGA-D6-6516-01	2027.263287	1278.67135	-IS_high	-SS_high
TCGA-D6-6517-01	-406.5892667	-1616.16073	-IS_low	-SS_low
TCGA-D6-6823-01	1555.580791	563.7450131	-IS_high	-SS_high
TCGA-D6-6824-01	2042.04429	446.5460714	-IS_high	-SS_high
TCGA-D6-6825-01	6.053597796	-1282.392445	-IS_low	-SS_low
TCGA-D6-6826-01	91.62077761	-1085.078175	-IS_low	-SS_low
TCGA-D6-6827-01	348.0846646	222.8918898	-IS_low	-SS_high
TCGA-D6-8568-01	1089.715673	983.5521302	-IS_high	-SS_high
TCGA-D6-8569-01	-648.5402037	-461.6746384	-IS_low	-SS_low
TCGA-D6-A4Z9-01	437.117102	-765.3678322	-IS_low	-SS_low
TCGA-D6-A4ZB-01	-88.36533504	-1037.039829	-IS_low	-SS_low
TCGA-D6-A6EK-01	2125.081612	-753.53875	-IS_high	-SS_low
TCGA-D6-A6EM-01	130.5918193	585.3071265	-IS_low	-SS_high
TCGA-D6-A6EN-01	1358.189965	-260.1088191	-IS_high	-SS_high
TCGA-D6-A6EO-01	-261.0766402	-1486.668488	-IS_low	-SS_low
TCGA-D6-A6EP-01	1330.379874	-584.665295	-IS_high	-SS_low
TCGA-D6-A6EQ-01	1619.962124	660.7789164	-IS_high	-SS_high
TCGA-D6-A6ES-01	472.1249007	300.7286303	-IS_low	-SS_high
TCGA-D6-A74Q-01	-179.1955114	-1199.108037	-IS_low	-SS_low
TCGA-DQ-5624-01	238.2412932	-1294.381019	-IS_low	-SS_low
TCGA-DQ-5625-01	1528.811261	1065.693795	-IS_high	-SS_high

TCGA-DQ-5630-01	1619.168688	568.3686989	-IS_high	-SS_high
TCGA-DQ-5631-01	449.8203605	312.5054027	-IS_low	-SS_high
TCGA-DQ-7591-01	1746.548971	-797.8455	-IS_high	-SS_low
TCGA-DQ-7592-01	1157.277934	-890.2988845	-IS_high	-SS_low
TCGA-F7-7848-01	1208.678939	-244.8269133	-IS_high	-SS_high
TCGA-F7-8298-01	2577.688996	1264.52808	-IS_high	-SS_high
TCGA-F7-8489-01	510.7815202	-1077.193465	-IS_low	-SS_low
TCGA-F7-A50G-01	677.2732377	-800.1962618	-IS_low	-SS_low
TCGA-F7-A50I-01	2082.8162	1173.689226	-IS_high	-SS_high
TCGA-F7-A50J-01	2912.765341	321.9398881	-IS_high	-SS_high
TCGA-F7-A61S-01	9.747991461	-1128.676263	-IS_low	-SS_low
TCGA-F7-A61V-01	2250.765573	303.5996902	-IS_high	-SS_high
TCGA-F7-A61W-01	1670.548045	-64.66477368	-IS_high	-SS_high
TCGA-F7-A620-01	227.7322753	-1278.493211	-IS_low	-SS_low
TCGA-F7-A622-01	1233.245192	-493.0457879	-IS_high	-SS_low
TCGA-F7-A623-01	1939.090634	-303.0373316	-IS_high	-SS_high
TCGA-F7-A624-01	1457.172711	797.7021215	-IS_high	-SS_high
TCGA-H7-7774-01	1008.728846	581.1875714	-IS_high	-SS_high
TCGA-H7-8501-01	1318.964607	504.383336	-IS_high	-SS_high
TCGA-H7-8502-01	1889.512666	520.8718314	-IS_high	-SS_high
TCGA-H7-A76A-01	1057.662251	182.0902495	-IS_high	-SS_high
TCGA-HD-7229-01	594.8096059	-497.0745894	-IS_low	-SS_low
TCGA-HD-7753-01	1107.14776	-52.09701606	-IS_high	-SS_high
TCGA-HD-7754-01	373.9797684	-911.6558322	-IS_low	-SS_low
TCGA-HD-7831-01	643.3034783	-174.7864872	-IS_low	-SS_high
TCGA-HD-7832-01	1960.037414	817.4401629	-IS_high	-SS_high
TCGA-HD-8224-01	789.837713	475.2491014	-IS_low	-SS_high
TCGA-HD-8314-01	885.2210257	-811.5039527	-IS_high	-SS_low
TCGA-HD-A4C1-01	1537.254788	-581.4425604	-IS_high	-SS_low
TCGA-HD-A633-01	1719.944256	-48.41688777	-IS_high	-SS_high
TCGA-HD-A634-01	616.7189066	387.6580538	-IS_low	-SS_high
TCGA-HD-A6HZ-01	2827.539861	1232.983299	-IS_high	-SS_high
TCGA-HD-A6I0-01	442.0334033	-971.555785	-IS_low	-SS_low
TCGA-HL-7533-01	1564.052347	620.0331263	-IS_high	-SS_high
TCGA-IQ-7630-01	409.1819119	-458.7667087	-IS_low	-SS_low
TCGA-IQ-7631-01	2089.301349	445.7589981	-IS_high	-SS_high
TCGA-IQ-7632-01	717.9816149	-110.0221897	-IS_low	-SS_high
TCGA-IQ-A61E-01	1695.931803	-397.4736642	-IS_high	-SS_low
TCGA-IQ-A61G-01	1709.681303	-210.6502271	-IS_high	-SS_high
TCGA-IQ-A61H-01	2305.925721	-25.35563079	-IS_high	-SS_high
TCGA-IQ-A61I-01	2014.54727	-315.1429464	-IS_high	-SS_low
TCGA-IQ-A61J-01	650.7821105	-717.5908252	-IS_low	-SS_low
TCGA-IQ-A61O-01	377.2973313	-38.42645621	-IS_low	-SS_high
TCGA-IQ-A6SH-01	270.2603331	57.49912545	-IS_low	-SS_high

TCGA-KU-A66S-01	746.2775806	-812.310098	-IS_low	-SS_low
TCGA-KU-A66T-01	763.6326629	-615.9379719	-IS_low	-SS_low
TCGA-KU-A6H7-01	28.23577473	-111.5813311	-IS_low	-SS_high
TCGA-KU-A6H8-01	1842.663994	716.442096	-IS_high	-SS_high
TCGA-MT-A51W-01	988.3192583	-103.3705769	-IS_high	-SS_high
TCGA-MT-A51X-01	621.2201145	-1096.334551	-IS_low	-SS_low
TCGA-MT-A67A-01	1007.117129	419.6506186	-IS_high	-SS_high
TCGA-MT-A67D-01	2433.879658	141.3073921	-IS_high	-SS_high
TCGA-MT-A67F-01	680.4975124	-169.0003797	-IS_low	-SS_high
TCGA-MT-A7BN-01	1757.062173	-1174.265582	-IS_high	-SS_low
TCGA-MZ-A5BI-01	697.3083494	-709.361133	-IS_low	-SS_low
TCGA-MZ-A6I9-01	1363.904846	961.5990074	-IS_high	-SS_high
TCGA-MZ-A7D7-01	163.1579463	-1134.746638	-IS_low	-SS_low
TCGA-P3-A5Q5-01	951.6390102	-568.1128395	-IS_high	-SS_low
TCGA-P3-A5Q6-01	1912.765538	716.7722086	-IS_high	-SS_high
TCGA-P3-A5QA-01	1115.07969	2.953307847	-IS_high	-SS_high
TCGA-P3-A5QE-01	132.3593086	-910.9166089	-IS_low	-SS_low
TCGA-P3-A5QF-01	2388.746816	235.5281544	-IS_high	-SS_high
TCGA-P3-A6SW-01	1738.538484	-410.0136712	-IS_high	-SS_low
TCGA-P3-A6SX-01	-370.2212874	-1550.628984	-IS_low	-SS_low
TCGA-P3-A6T0-01	1354.441418	-1074.671912	-IS_high	-SS_low
TCGA-P3-A6T2-01	1009.384398	-221.6964101	-IS_high	-SS_high
TCGA-P3-A6T3-01	895.0381739	-558.8567598	-IS_high	-SS_low
TCGA-P3-A6T4-01	1247.934053	-476.0307059	-IS_high	-SS_low
TCGA-P3-A6T5-01	609.8327476	67.61081033	-IS_low	-SS_high
TCGA-P3-A6T6-01	659.7420964	-227.355719	-IS_low	-SS_high
TCGA-P3-A6T7-01	1264.011239	-253.7707579	-IS_high	-SS_high
TCGA-P3-A6T8-01	1605.753508	-450.979511	-IS_high	-SS_low
TCGA-QK-A64Z-01	1841.456411	1320.589907	-IS_high	-SS_high
TCGA-QK-A652-01	1066.213229	-154.9937726	-IS_high	-SS_high
TCGA-QK-A6IF-01	-423.8708449	-1660.718695	-IS_low	-SS_low
TCGA-QK-A6IG-01	654.3884904	-709.0183915	-IS_low	-SS_low
TCGA-QK-A6IH-01	971.0813171	-626.3797808	-IS_high	-SS_low
TCGA-QK-A6II-01	1170.272706	8.305259855	-IS_high	-SS_high
TCGA-QK-A6IJ-01	160.1414389	-1157.242578	-IS_low	-SS_low
TCGA-QK-A6V9-01	228.3958571	-800.8451192	-IS_low	-SS_low
TCGA-QK-A6VB-01	817.2009809	-62.04530748	-IS_low	-SS_high
TCGA-QK-A6VC-01	343.610763	-618.7913988	-IS_low	-SS_low
TCGA-QK-A8Z7-01	1141.304583	-602.5390523	-IS_high	-SS_low
TCGA-QK-A8Z8-01	2169.333629	178.4620693	-IS_high	-SS_high
TCGA-QK-A8Z9-01	712.7781487	-604.1347254	-IS_low	-SS_low
TCGA-QK-A8ZA-01	-30.62890453	-572.7112397	-IS_low	-SS_low
TCGA-QK-A8ZB-01	1676.42843	-328.5943369	-IS_high	-SS_low
TCGA-QK-AA3J-01	1246.758962	-264.9118198	-IS_high	-SS_high

TCGA-QK-AA3K-01	1967.838894	351.4761616	-IS_high	-SS_high
TCGA-RS-A6TO-01	600.5277301	-430.4604102	-IS_low	-SS_low
TCGA-RS-A6TP-01	1336.17975	1084.796117	-IS_high	-SS_high
TCGA-T2-A6WX-01	586.9359042	675.7249496	-IS_low	-SS_high
TCGA-T2-A6WZ-01	1050.645762	-467.6794257	-IS_high	-SS_low
TCGA-T2-A6X0-01	1380.073843	1212.380174	-IS_high	-SS_high
TCGA-T2-A6X2-01	862.1355331	458.4561945	-IS_low	-SS_high
TCGA-T3-A92M-01	1453.249398	-93.5979839	-IS_high	-SS_high
TCGA-T3-A92N-01	2016.471716	-264.259492	-IS_high	-SS_high
TCGA-TN-A7HI-01	-182.8025187	-1056.690578	-IS_low	-SS_low
TCGA-TN-A7HJ-01	876.7952682	-868.6027626	-IS_low	-SS_low
TCGA-TN-A7HL-01	977.3569994	-98.87723786	-IS_high	-SS_high
TCGA-UF-A718-01	1632.046788	614.326783	-IS_high	-SS_high
TCGA-UF-A719-01	-1003.149592	-1778.731564	-IS_low	-SS_low
TCGA-UF-A71A-01	-455.9380395	253.2896626	-IS_low	-SS_high
TCGA-UF-A71B-01	588.7884742	-609.0200967	-IS_low	-SS_low
TCGA-UF-A71E-01	202.6224939	-199.3906642	-IS_low	-SS_high
TCGA-UF-A7J9-01	1430.017512	-558.5295903	-IS_high	-SS_low
TCGA-UF-A7JA-01	-175.6614732	-168.6623047	-IS_low	-SS_high
TCGA-UF-A7JC-01	-12.42309365	195.5559082	-IS_low	-SS_high
TCGA-UF-A7JD-01	2284.202739	1259.513714	-IS_high	-SS_high
TCGA-UF-A7JF-01	1646.101857	262.2015317	-IS_high	-SS_high
TCGA-UF-A7JH-01	1782.380384	466.9131228	-IS_high	-SS_high
TCGA-UF-A7JJ-01	1373.131237	-1033.697443	-IS_high	-SS_low
TCGA-UF-A7JK-01	-340.0653815	-491.416433	-IS_low	-SS_low
TCGA-UF-A7JO-01	60.44630687	-611.264205	-IS_low	-SS_low
TCGA-UF-A7JS-01	432.6475307	-281.9727811	-IS_low	-SS_high
TCGA-UF-A7JT-01	603.2879718	-1060.291562	-IS_low	-SS_low
TCGA-UP-A6WW-01	963.5676045	-105.9766073	-IS_high	-SS_high
TCGA-WA-A7H4-01	-91.02108375	-1251.526894	-IS_low	-SS_low
