# Supplementary material for: Immune Infiltration-Related ceRNA Network Revealing Potential Biomarkers for Prognosis of Head and Neck Squamous Cell Carcinoma
Source: Dis Markers. 2022 Sep 2;2022:1014347. doi: 10.1155/2022/1014347 (PMC9463596; doi:10.1155/2022/1014347)
Supplement: Supplementary 2 — Table S2. Immune scores and stromal scores of HNSCC patients. [file 1014347.f2.pdf]

Table S1 Clinical characteristic of HNSCC patients.

Patients	Age at initial pathologic diagnosis	Gender	Clinical_stage	Metastasis	N stage	T stage	Lymph node examined count	Neoplasm histologic grade	Primary therapy outcome success	Targeted molecular therapy	Outcome	Time
TCGA-4P-AA8J	66.0	male	stage iva	m0	n2a	t4a	44.0	g2	complete remission/response	no	0	102
TCGA-BA-4074	69.0	male	stage iva	m0	n2c	t3	47.0	g3	complete remission/response	yes	1	462
TCGA-BA-4076	39.0	male	stage iva	m0	n2c	t3	NA	g2	complete remission/response	yes	1	415
TCGA-BA-4078	83.0	male	stage iva	m0	n2a	t2	52.0	g2	NA	no	1	276
TCGA-BA-5151	72.0	male	stage iva	m0	n0	t4a	33.0	g1	complete remission/response	no	0	722
TCGA-BA-5152	56.0	male	stage iva	m0	n0	t4a	28.0	g2	complete remission/response	no	0	1288
TCGA-BA-5153	51.0	male	stage iii	m0	n1	t2	0.0	g2	complete remission/response	no	1	1762
TCGA-BA-5555	54.0	male	stage iva	m0	n2c	t2	68.0	g2	complete remission/response	no	0	520
TCGA-BA-5556	58.0	female	stage ii	m0	n0	t2	81.0	g3	complete remission/response	no	0	725

TCGA-BA-5558	65.0	male	stage iva	m0	n1	t4a	37.0	g1	complete remission/response	no	0	199 5
TCGA-BA-6868	53.0	male	stage ivb	m0	n3	t3	NA	g2	partial remission/response	yes	1	472
TCGA-BA-6869	62.0	male	stage iii	m0	n0	t3	80.0	g2	complete remission/response	no	0	644
TCGA-BA-6870	60.0	female	stage ivc	m1	n2c	t3	NA	g2	NA	yes	1	451
TCGA-BA-6872	47.0	male	stage iva	m0	n2c	t3	50.0	g2	NA	yes	1	384
TCGA-BA-6873	28.0	male	stage iva	m0	n2b	t2	48.0	g2	progressive disease	no	0	122
TCGA-BA-7269	61.0	male	stage iii	m0	n1	t2	17.0	g1	complete remission/response	no	0	127 3
TCGA-BA-A4IF	59.0	male	stage iva	m0	n0	t4	NA	g2	complete remission/response	yes	0	895
TCGA-BA-A4IH	57.0	male	stage iva	m0	n2b	t2	39.0	g3	complete remission/response	yes	0	622
TCGA-BA-A6D8	59.0	male	stage iva	m0	n2c	t4a	77.0	g2	complete remission/response	yes	0	850
TCGA-BA-A6DA	41.0	female	stage iva	m0	n2c	t4a	87.0	g2	complete remission/response	NA	0	351
TCGA-BA-A6DB	24.0	female	stage ii	m0	n0	t2	24.0	g1	complete remission/response	no	0	216
TCGA-BA-A6DD	44.0	male	stage iva	m0	n2c	t4a	42.0	g2	progressive disease	NA	1	173

TCGA-BA-A6DE	70.0	female	stage iii	m0	n0	t3	73.0	g2	complete remission/response	NA	0	440
TCGA-BA-A6DG	49.0	male	stage iva	m0	n2b	t4a	NA	g2	progressive disease	NA	1	69
TCGA-BA-A6DI	62.0	male	stage iii	m0	n0	t3	NA	g2	complete remission/response	NA	1	336
TCGA-BA-A6DJ	62.0	male	stage iva	m0	n2b	t4	101.0	g2	complete remission/response	yes	1	407
TCGA-BA-A6DL	59.0	male	stage iii	m0	n0	t3	NA	g2	complete remission/response	yes	0	623
TCGA-BA-A8YP	50.0	male	stage ivb	m0	n3	t4	66.0	g2	complete remission/response	yes	0	499
TCGA-BB-4217	68.0	male	stage iva	m0	n2c	t4a	51.0	g3	partial remission/response	no	0	187
TCGA-BB-4223	48.0	male	stage iva	m0	n2b	t3	NA	g3	complete remission/response	yes	0	322 1
TCGA-BB-4224	52.0	male	stage iii	m0	n0	t3	51.0	g2	progressive disease	no	0	278
TCGA-BB-4225	73.0	male	stage iva	m0	n2c	t3	NA	g3	partial remission/response	yes	0	146
TCGA-BB-4227	66.0	male	stage iva	m0	n2b	t4a	25.0	g2	progressive disease	no	0	134
TCGA-BB-4228	50.0	male	stage ii	m0	n0	t2	NA	gx	complete remission/response	no	0	559
TCGA-BB-8596	69.0	female	stage iva	m0	n1	t4	NA	g3	complete remission/response	no	0	216 1
TCGA-BB-8601	84.0	male	stage ii	m0	n0	t2	16.0	g2	progressive disease	no	0	624

TCGA-BB-A5HY	64.0	male	stage iva	m0	n2b	t3	24.0	g3	complete remission/response	yes	1	321
TCGA-BB-A5HZ	65.0	male	stage iva	m0	n1	t4	39.0	g2	complete remission/response	yes	0	827
TCGA-BB-A6UO	61.0	female	stage iva	mx	n2	t3	28.0	g2	complete remission/response	NA	1	268
TCGA-C9-A47Z	72.0	female	stage iii	m0	n1	t2	2.0	g1	complete remission/response	no	1	191
TCGA-C9-A480	45.0	female	stage iii	m0	n0	t3	NA	g1	complete remission/response	no	0	386
TCGA-CN-4722	61.0	female	stage ii	m0	n0	t2	53.0	g1	complete remission/response	no	0	148 3
TCGA-CN-4723	67.0	male	stage iva	m0	n0	t4a	55.0	g2	complete remission/response	no	0	169 9
TCGA-CN-4725	60.0	male	stage ii	m0	n0	t2	16.0	g2	complete remission/response	no	0	115 7
TCGA-CN-4726	68.0	male	stage iva	m0	n2	t3	14.0	g2	progressive disease	no	1	142
TCGA-CN-4728	56.0	male	stage iva	m0	n2	t2	58.0	g2	complete remission/response	yes	0	172 4
TCGA-CN-4729	73.0	male	stage iva	m0	n0	t4a	24.0	g2	complete remission/response	no	0	392
TCGA-CN-4730	62.0	male	stage iva	m0	n2b	t4a	37.0	g2	complete remission/response	no	0	817
TCGA-CN-4731	63.0	female	stage iva	m0	n2	t4a	52.0	g3	progressive disease	no	1	998

TCGA-CN-4733	61.0	male	stage i	m0	n0	t1	29.0	g3	complete remission/response	no	0	1586
TCGA-CN-4735	52.0	male	stage iva	m0	n2b	t3	70.0	g3	complete remission/response	yes	0	1737
TCGA-CN-4736	70.0	female	stage ii	m0	n0	t2	NA	g2	complete remission/response	no	1	395
TCGA-CN-4737	19.0	male	stage ii	m0	n0	t2	36.0	g2	complete remission/response	yes	0	625
TCGA-CN-4738	53.0	male	stage iva	m0	n0	t4a	39.0	g2	NA	NA	1	436
TCGA-CN-4739	71.0	male	stage iva	m0	n0	t4a	54.0	g2	complete remission/response	yes	1	1394
TCGA-CN-4740	79.0	female	stage iva	m0	n0	t4a	26.0	g2	complete remission/response	no	1	839
TCGA-CN-4741	75.0	male	stage iva	m0	n0	t4a	46.0	g2	complete remission/response	yes	0	2239
TCGA-CN-4742	48.0	female	stage iva	m0	n1	t4a	49.0	g3	progressive disease	yes	1	397
TCGA-CN-5355	64.0	male	stage iva	m0	n0	t4a	27.0	g2	complete remission/response	no	0	1278
TCGA-CN-5356	56.0	male	stage iii	m0	n0	t3	97.0	g2	complete remission/response	no	0	1409
TCGA-CN-5358	60.0	male	stage iii	m0	n1	t2	57.0	g2	complete remission/response	no	1	261
TCGA-CN-5359	59.0	male	stage iva	m0	n1	t4a	29.0	g2	complete remission/response	yes	1	377

TCGA-CN-5360	68.0	male	stage iva	m0	n0	t4a	54.0	g2	complete remission/response	yes	0	2169
TCGA-CN-5361	80.0	male	NA	NA	nx	tx	56.0	g2	complete remission/response	no	1	2120
TCGA-CN-5363	48.0	male	stage ivb	m0	n3	t4a	28.0	g3	complete remission/response	NA	1	253
TCGA-CN-5364	55.0	male	stage iva	m0	n2c	t4a	73.0	g2	complete remission/response	yes	1	493
TCGA-CN-5365	38.0	male	stage ivc	m1	n2	t1	9.0	g2	progressive disease	NA	1	351
TCGA-CN-5366	51.0	male	stage iva	m0	n2c	t4a	47.0	g2	complete remission/response	yes	1	360
TCGA-CN-5367	60.0	female	stage iva	m0	n0	t4a	46.0	g2	complete remission/response	yes	1	352
TCGA-CN-5369	90.0	female	stage iva	m0	n1	t4a	82.0	g3	complete remission/response	no	1	380
TCGA-CN-5370	78.0	male	stage ii	m0	n0	t2	40.0	g3	complete remission/response	NA	1	259
TCGA-CN-5373	55.0	female	stage ii	m0	n0	t2	30.0	g1	complete remission/response	no	0	1584
TCGA-CN-5374	56.0	female	stage iva	m0	n2	t2	NA	g3	complete remission/response	yes	1	1732
TCGA-CN-6010	53.0	male	stage iva	m0	n0	t4a	39.0	g2	complete remission/response	yes	0	1523
TCGA-CN-6011	57.0	male	stage iva	m0	n0	t4a	44.0	g3	complete remission/response	yes	0	933

TCGA-CN-6012	66.0	male	stage iii	m0	n1	t3	35.0	g2	complete remission/response	yes	0	1460
TCGA-CN-6013	56.0	male	stage iva	m0	n2b	t4a	28.0	g3	complete remission/response	yes	1	727
TCGA-CN-6016	64.0	male	stage iva	m0	n1	t4a	75.0	g2	complete remission/response	yes	0	1443
TCGA-CN-6017	55.0	male	stage iii	m0	n1	t2	30.0	g2	complete remission/response	yes	1	853
TCGA-CN-6018	85.0	female	stage iva	m0	n2c	t4a	20.0	g2	complete remission/response	no	1	580
TCGA-CN-6019	61.0	male	stage iva	m0	n0	t4a	45.0	g2	complete remission/response	yes	0	1038
TCGA-CN-6020	58.0	male	stage iva	m0	n2c	t3	68.0	g2	complete remission/response	no	1	205
TCGA-CN-6021	63.0	female	stage iii	m0	n0	t3	NA	g2	progressive disease	NA	1	276
TCGA-CN-6022	49.0	male	stage iva	m0	n1	t4a	33.0	g3	progressive disease	no	1	281
TCGA-CN-6023	73.0	male	stage iva	m0	n0	t4a	31.0	gx	complete remission/response	yes	0	1584
TCGA-CN-6024	66.0	male	stage iva	m0	n2	t4a	39.0	g2	progressive disease	NA	1	337
TCGA-CN-6988	47.0	male	stage iva	m0	n2b	t4a	81.0	g3	complete remission/response	NA	0	318
TCGA-CN-6989	64.0	male	stage iva	m0	n2b	t3	56.0	g2	complete remission/response	yes	1	980
TCGA-CN-6994	67.0	male	stage	m0	n2b	t4a	37.0	g2	complete	no	0	118

			iva						remission/response			3
TCGA-CN-6995	78.0	male	stage iva	m0	n1	t4a	51.0	g2	complete remission/response	no	1	112
TCGA-CN-6996	58.0	female	stage iva	m0	n2b	t3	24.0	g2	progressive disease	yes	1	530
TCGA-CN-6998	53.0	male	stage iva	m0	n1	t4a	26.0	g2	progressive disease	yes	1	357
TCGA-CN-A497	63.0	male	stage iva	m0	n2c	t2	69.0	g2	complete remission/response	no	0	1065
TCGA-CN-A498	61.0	female	stage ii	m0	n0	t2	23.0	g1	complete remission/response	yes	1	773
TCGA-CN-A499	60.0	female	stage i	m0	n0	t1	37.0	g2	complete remission/response	no	0	717
TCGA-CN-A49A	60.0	male	stage iva	m0	n0	t4a	59.0	g2	complete remission/response	yes	1	526
TCGA-CN-A49B	71.0	male	stage iii	m0	n1	t3	39.0	g3	complete remission/response	no	0	904
TCGA-CN-A49C	67.0	male	stage iva	m0	n2b	t4a	NA	gx	complete remission/response	yes	0	645
TCGA-CN-A63T	60.0	male	stage iva	m0	n2b	t3	80.0	g3	complete remission/response	yes	0	225
TCGA-CN-A63U	50.0	male	stage iii	m0	n0	t3	45.0	g3	complete remission/response	no	0	964
TCGA-CN-A63V	59.0	male	stage iva	m0	n0	t4	45.0	g2	complete remission/response	no	0	679
TCGA-CN-A63W	48.0	female	stage	m0	n2c	t4a	66.0	g2	complete	yes	1	377

			iva						remission/response			
TCGA-CN-A641	47.0	male	stage iva	m0	n2c	t4	70.0	g2	complete remission/response	yes	0	367
TCGA-CN-A642	57.0	male	stage iva	m0	n2c	t3	110.0	g3	progressive disease	no	1	82
TCGA-CN-A6UY	57.0	male	stage iva	m0	n2a	t3	43.0	g2	complete remission/response	yes	0	713
TCGA-CN-A6V6	59.0	male	stage iva	m0	n2b	t2	33.0	gx	complete remission/response	yes	0	635
TCGA-CN-A6V7	40.0	male	stage iva	m0	n2b	t2	NA	gx	complete remission/response	yes	0	594
TCGA-CQ-5324	59.0	male	stage iva	m0	n2a	t3	24.0	g2	complete remission/response	no	0	1593
TCGA-CQ-5325	65.0	male	stage i	m0	n0	t1	22.0	g2	complete remission/response	no	1	654
TCGA-CQ-5326	67.0	male	stage iva	m0	n2b	t4a	56.0	g3	progressive disease	no	1	89
TCGA-CQ-5327	61.0	female	stage iva	m0	n2c	t3	55.0	g2	complete remission/response	yes	0	1660
TCGA-CQ-5329	46.0	female	stage iii	m0	n1	t3	29.0	g2	complete remission/response	no	0	2143
TCGA-CQ-5330	69.0	female	stage iii	m0	n1	t3	44.0	g3	complete remission/response	yes	0	1897
TCGA-CQ-5331	73.0	female	stage iva	m0	n0	t4a	NA	g2	complete remission/response	no	0	1399
TCGA-CQ-5332	87.0	male	stage iii	m0	n0	t3	31.0	g2	NA	NA	1	317

TCGA-CQ-5333	74.0	male	stage i	m0	n0	t1	21.0	g3	complete remission/response	no	1	341
TCGA-CQ-5334	87.0	male	stage ivb	m0	n2b	t4b	46.0	g2	progressive disease	no	1	129
TCGA-CQ-6218	52.0	female	stage iii	m0	n0	t3	15.0	g2	complete remission/response	yes	0	1253
TCGA-CQ-6219	50.0	female	stage iva	m0	n2b	t3	55.0	g2	complete remission/response	no	1	479
TCGA-CQ-6220	69.0	male	stage iii	m0	n1	t2	42.0	g2	complete remission/response	no	1	985
TCGA-CQ-6222	63.0	male	stage iva	m0	n2b	t3	66.0	g2	complete remission/response	yes	0	2016
TCGA-CQ-6223	69.0	male	stage iva	m0	n0	t4a	32.0	g2	complete remission/response	no	0	1428
TCGA-CQ-6224	52.0	male	stage ii	m0	n0	t2	29.0	g3	complete remission/response	yes	0	1721
TCGA-CQ-6225	65.0	male	stage ii	m0	n0	t2	25.0	g2	persistent disease	no	1	403
TCGA-CQ-6227	77.0	male	stage iii	m0	n1	t3	59.0	g2	progressive disease	no	1	129
TCGA-CQ-6228	71.0	female	stage iii	m0	n1	t2	76.0	g2	complete remission/response	no	1	456
TCGA-CQ-6229	61.0	male	stage iii	m0	n1	t2	21.0	g2	complete remission/response	no	0	1179
TCGA-CQ-7065	40.0	male	stage ii	m0	n0	t2	39.0	g2	complete remission/response	no	0	1628
TCGA-CQ-7069	77.0	female	stage ii	m0	n0	t2	28.0	g2	complete remission/response	no	0	1274

TCGA-CQ-7071	76.0	female	stage iva	m0	n1	t4a	22.0	g2	complete remission/response	no	0	131 1
TCGA-CQ-7072	51.0	male	stage iva	m0	n0	t4a	28.0	g3	complete remission/response	no	0	235 9
TCGA-CQ-A4C6	63.0	male	stage ii	m0	n0	t2	23.0	g2	complete remission/response	no	0	135 3
TCGA-CQ-A4C9	56.0	male	NA	mx	nx	tx	47.0	g2	complete remission/response	no	0	707
TCGA-CQ-A4CA	NA	male	stage ii	m0	n0	t2	25.0	g2	complete remission/response	NA	0	
TCGA-CQ-A4CB	59.0	male	stage ii	m0	n0	t2	26.0	g2	complete remission/response	no	0	893
TCGA-CQ-A4CD	69.0	male	stage ivb	m0	n0	t4b	47.0	g3	complete remission/response	no	0	102 2
TCGA-CQ-A4CE	76.0	female	stage ii	m0	n0	t2	37.0	g2	complete remission/response	no	0	897
TCGA-CQ-A4CG	78.0	female	stage ii	m0	n0	t2	16.0	g2	complete remission/response	no	1	430
TCGA-CQ-A4CH	58.0	male	stage iva	m0	n2c	t4a	55.0	g2	complete remission/response	yes	1	379
TCGA-CQ-A4CI	73.0	male	NA	mx	nx	tx	49.0	g3	complete remission/response	no	0	950
TCGA-CR-5243	51.0	male	stage iva	m0	n2b	t3	NA	g3	complete remission/response	yes	0	256 2
TCGA-CR-5247	48.0	male	stage iii	m0	n1	t3	NA	g2	complete remission/response	yes	0	358

TCGA-CR-5248	53.0	male	stage iva	m0	n2c	t4a	NA	g3	complete remission/response	yes	0	1663
TCGA-CR-5249	35.0	female	stage ii	m0	n0	t2	NA	gx	complete remission/response	yes	0	1152
TCGA-CR-5250	71.0	male	stage ii	m0	n0	t2	NA	g3	complete remission/response	yes	0	799
TCGA-CR-6467	59.0	male	stage iva	m0	n2b	t2	41.0	g3	complete remission/response	no	0	1777
TCGA-CR-6470	38.0	male	stage iva	m0	n2c	t1	24.0	g2	complete remission/response	yes	0	1521
TCGA-CR-6471	58.0	male	stage iva	m0	n0	t4a	18.0	g2	complete remission/response	no	1	1202
TCGA-CR-6473	68.0	male	stage iva	m0	n2b	t3	NA	g3	complete remission/response	yes	0	1125
TCGA-CR-6474	51.0	male	stage iva	m0	n2a	t3	31.0	g2	complete remission/response	no	1	564
TCGA-CR-6477	56.0	female	stage iva	m0	n2b	t3	NA	g2	complete remission/response	NA	0	514
TCGA-CR-6480	53.0	male	stage iva	m0	n2a	t2	NA	g2	complete remission/response	NA	0	362
TCGA-CR-6481	47.0	male	stage iva	m0	n2a	t2	NA	g2	complete remission/response	NA	0	311
TCGA-CR-6482	62.0	male	stage iva	m0	n2b	t2	NA	g3	complete remission/response	NA	0	345
TCGA-CR-6484	67.0	female	stage iva	m0	n0	t4a	22.0	g2	complete remission/response	yes	0	354

TCGA-CR-6487	50.0	male	stage ii	m0	n0	t2	NA	g3	complete remission/response	no	0	234
TCGA-CR-6488	68.0	female	stage ii	m0	n0	t2	22.0	g2	complete remission/response	no	0	379
TCGA-CR-6491	60.0	male	stage iva	m0	nx	t4a	49.0	g2	complete remission/response	yes	0	693
TCGA-CR-6492	78.0	male	stage iva	m0	n1	t4a	37.0	g3	complete remission/response	no	0	479
TCGA-CR-6493	69.0	male	stage iva	m0	n2b	t3	16.0	g2	complete remission/response	yes	1	282
TCGA-CR-7364	66.0	male	stage iii	m0	n0	t3	36.0	g2	complete remission/response	no	0	143 5
TCGA-CR-7365	60.0	male	stage iva	m0	n2b	t4a	45.0	g2	complete remission/response	yes	0	119 1
TCGA-CR-7367	52.0	male	stage ivb	m0	n3	t4a	30.0	g1	complete remission/response	yes	0	144 0
TCGA-CR-7368	54.0	male	stage iva	m0	n0	t4a	41.0	g2	complete remission/response	yes	0	124 5
TCGA-CR-7369	59.0	male	stage iva	m0	n0	t4a	23.0	g2	complete remission/response	no	1	109 0
TCGA-CR-7370	72.0	female	stage ii	m0	n0	t2	NA	g2	complete remission/response	no	0	105
TCGA-CR-7371	45.0	female	stage iii	m0	n0	t3	104.0	g2	NA	no	1	94
TCGA-CR-7372	45.0	male	stage ii	m0	n0	t2	19.0	g1	complete remission/response	no	0	759
TCGA-CR-7373	66.0	male	stage	m0	n2b	t4a	19.0	g2	complete	yes	0	889

			iva						remission/response			
TCGA-CR-7374	67.0	female	stage ii	m0	n0	t2	NA	g2	NA	yes	0	30
TCGA-CR-7376	83.0	male	stage ii	m0	n0	t2	22.0	g2	complete remission/response	no	0	972
TCGA-CR-7377	58.0	male	stage iva	m0	n1	t4a	29.0	g3	complete remission/response	no	1	279
TCGA-CR-7379	78.0	female	stage iva	m0	n2b	t4a	33.0	g2	complete remission/response	no	0	103 6
TCGA-CR-7380	58.0	male	stage iii	m0	n0	t3	17.0	g2	complete remission/response	yes	1	606
TCGA-CR-7382	49.0	male	stage iva	m0	n2c	t2	83.0	g2	complete remission/response	yes	0	796
TCGA-CR-7383	79.0	female	stage i	m0	n0	t1	21.0	g2	complete remission/response	no	1	521
TCGA-CR-7385	42.0	male	stage iva	m0	n2b	t2	NA	g2	complete remission/response	yes	0	997
TCGA-CR-7386	69.0	male	stage iva	m0	n1	t4a	47.0	g1	complete remission/response	yes	0	143 0
TCGA-CR-7388	70.0	female	stage iva	m0	n2	t2	NA	g2	complete remission/response	yes	1	823
TCGA-CR-7389	55.0	male	stage iii	m0	n1	t2	NA	g2	complete remission/response	yes	0	392
TCGA-CR-7390	67.0	male	stage iii	m0	n0	t3	16.0	g2	complete remission/response	no	0	150 8
TCGA-CR-7391	36.0	female	stage i	m0	n0	t1	10.0	g1	complete remission/response	no	0	913

TCGA-CR-7392	67.0	female	stage iva	m0	n2b	t3	36.0	g1	complete remission/response	no	0	142 5
TCGA-CR-7393	26.0	male	stage i	m0	n0	t1	44.0	g2	complete remission/response	yes	0	993
TCGA-CR-7394	70.0	male	stage iva	m0	n2c	t3	57.0	g2	complete remission/response	no	0	134 6
TCGA-CR-7395	80.0	female	stage ii	m0	n0	t2	NA	g2	complete remission/response	no	0	930
TCGA-CR-7397	44.0	male	stage iva	m0	n2b	t3	34.0	g2	complete remission/response	yes	0	754
TCGA-CR-7398	53.0	female	stage ii	m0	n0	t2	23.0	g2	complete remission/response	no	0	156
TCGA-CR-7399	60.0	female	stage iva	m0	n2c	t4a	57.0	g3	complete remission/response	yes	0	181
TCGA-CR-7401	64.0	male	stage i	m0	n0	t1	27.0	g2	complete remission/response	no	0	107 7
TCGA-CR-7402	68.0	male	stage iii	m0	n0	t3	NA	g2	complete remission/response	yes	0	911
TCGA-CR-7404	53.0	male	stage iva	m0	n2b	t3	NA	g2	complete remission/response	yes	0	147 2
TCGA-CV-5430	61.0	male	stage iva	m0	n2b	t4a	9.0	g3	complete remission/response	no	0	424 1
TCGA-CV-5431	73.0	male	stage iva	m0	n2	t3	29.0	g3	complete remission/response	no	1	522
TCGA-CV-5432	68.0	male	stage iii	m0	n0	t3	65.0	g3	NA	no	0	393 0

TCGA-CV-5434	60.0	male	stage iva	m0	n1	t4a	30.0	g2	complete remission/response	no	1	331 4
TCGA-CV-5435	57.0	male	stage iva	m0	n0	t4a	66.0	g3	NA	yes	1	231 9
TCGA-CV-5436	65.0	male	stage iva	m0	n2b	t3	51.0	g2	persistent disease	no	1	584
TCGA-CV-5439	62.0	male	stage ii	m0	n0	t2	33.0	g2	NA	no	1	546
TCGA-CV-5440	52.0	male	stage iva	m0	n2b	t4a	47.0	g3	complete remission/response	no	0	327 0
TCGA-CV-5441	58.0	male	stage iva	m0	n1	t4a	62.0	g3	complete remission/response	no	0	288 6
TCGA-CV-5442	76.0	female	stage iva	m0	n2b	t4a	33.0	g3	complete remission/response	no	0	232 7
TCGA-CV-5443	63.0	male	stage iii	m0	n1	t3	11.0	g3	NA	no	0	278 4
TCGA-CV-5444	64.0	male	stage iva	m0	n0	t4a	21.0	g3	NA	no	0	243 7
TCGA-CV-5966	63.0	female	stage iva	m0	n1	t4a	23.0	g3	complete remission/response	no	1	545
TCGA-CV-5970	59.0	male	stage iva	m0	n2b	t4a	50.0	g2	NA	NA	1	406
TCGA-CV-5971	60.0	male	stage iva	m0	n2b	t2	26.0	g2	NA	no	0	701
TCGA-CV-5973	62.0	female	stage iii	m0	n0	t3	58.0	g3	complete remission/response	no	0	264 1
TCGA-CV-5976	50.0	male	stage iii	m0	n1	t3	50.0	g2	NA	NA	0	147

												8
TCGA-CV-5977	66.0	male	stage iii	m0	n1	t3	82.0	g2	complete remission/response	no	0	1840
TCGA-CV-5978	53.0	female	stage ivb	m0	n3	t4a	55.0	g2	NA	NA	1	215
TCGA-CV-5979	26.0	male	stage iii	m0	n1	t3	55.0	g2	complete remission/response	no	0	1315
TCGA-CV-6003	50.0	female	stage ii	m0	n0	t2	23.0	g2	NA	no	0	1665
TCGA-CV-6433	57.0	male	stage ii	m0	n0	t2	5.0	g3	complete remission/response	yes	0	641
TCGA-CV-6436	62.0	male	stage iii	m0	n0	t3	40.0	g1	NA	no	0	1899
TCGA-CV-6441	60.0	male	stage iii	m0	n0	t3	44.0	g3	progressive disease	no	1	292
TCGA-CV-6933	53.0	male	stage iva	m0	n0	t4a	32.0	g2	NA	no	1	2741
TCGA-CV-6934	66.0	female	stage iva	m0	n2b	t3	19.0	g2	complete remission/response	no	1	65
TCGA-CV-6935	67.0	male	stage iii	m0	n0	t3	4.0	g3	NA	NA	1	295
TCGA-CV-6936	68.0	male	stage iva	m0	n2b	t4a	10.0	g2	progressive disease	no	1	166
TCGA-CV-6937	71.0	male	stage ii	m0	n0	t2	NA	g2	NA	NA	1	624
TCGA-CV-6938	87.0	male	stage ii	m0	n0	t2	NA	g1	NA	NA	1	144
TCGA-CV-6939	60.0	male	stage iva	m0	n1	t4a	12.0	g3	NA	NA	1	666
TCGA-CV-6940	80.0	female	stage i	m0	n0	t1	12.0	g2	complete	no	1	804

									remission/response			
TCGA-CV-6941	51.0	male	stage iii	m0	n0	t3	29.0	g2	complete remission/response	no	1	342
TCGA-CV-6942	73.0	female	stage ii	m0	n0	t2	NA	g3	complete remission/response	no	0	428<br>2
TCGA-CV-6943	74.0	male	stage iii	m0	n0	t3	NA	g2	NA	NA	1	602
TCGA-CV-6945	41.0	male	stage iva	m0	n2b	t4a	45.0	g2	NA	NA	1	366
TCGA-CV-6948	79.0	female	stage iva	m0	n0	t4a	44.0	g2	NA	no	1	128<br>9
TCGA-CV-6950	64.0	male	stage iva	m0	n2c	t3	45.0	g2	complete remission/response	no	1	459
TCGA-CV-6951	57.0	male	stage iva	m0	n2c	t4a	29.0	g2	NA	NA	1	915
TCGA-CV-6952	65.0	female	stage iii	m0	n0	t3	9.0	g1	NA	no	1	185
TCGA-CV-6953	80.0	female	stage iii	m0	n0	t3	33.0	g1	complete remission/response	no	1	164<br>1
TCGA-CV-6954	59.0	male	stage iva	m0	n1	t4a	22.0	g2	complete remission/response	no	1	200<br>2
TCGA-CV-6955	74.0	female	stage ii	m0	n0	t2	NA	g3	NA	NA	1	334
TCGA-CV-6956	67.0	male	stage iva	m0	n2c	t4a	27.0	g2	complete remission/response	no	1	217
TCGA-CV-6959	48.0	male	stage iii	m0	n1	t3	28.0	g2	NA	no	1	256
TCGA-CV-6960	49.0	male	stage iii	m0	n0	t3	22.0	g3	NA	no	1	862
TCGA-CV-6961	61.0	male	stage ii	m0	n0	t2	NA	g3	complete remission/response	no	1	76

TCGA-CV-6962	65.0	male	stage iii	m0	n0	t3	NA	g2	NA	NA	1	126
TCGA-CV-7089	74.0	male	stage iva	m0	n2	t4a	22.0	g2	NA	NA	1	1972
TCGA-CV-7090	39.0	male	stage ii	m0	n0	t2	NA	g2	complete remission/response	no	0	5252
TCGA-CV-7091	54.0	male	stage i	m0	n0	t1	25.0	g3	NA	NA	0	3381
TCGA-CV-7095	87.0	female	stage iva	m0	n2a	t1	8.0	g2	NA	no	1	572
TCGA-CV-7097	53.0	male	stage iii	m0	n0	t3	NA	g3	NA	NA	1	385
TCGA-CV-7099	85.0	female	stage ii	m0	n0	t2	NA	g2	complete remission/response	no	1	243
TCGA-CV-7100	66.0	male	stage ii	m0	n0	t2	NA	g3	NA	NA	1	274
TCGA-CV-7101	80.0	male	stage ii	m0	n0	t2	0.0	g2	NA	NA	1	160
TCGA-CV-7102	76.0	female	stage iii	m0	n1	t3	24.0	g3	complete remission/response	no	1	56
TCGA-CV-7103	49.0	male	stage ii	m0	n0	t2	14.0	g2	NA	NA	1	1591
TCGA-CV-7104	61.0	female	stage iva	m0	n2	t2	30.0	g2	complete remission/response	no	1	393
TCGA-CV-7177	82.0	female	stage i	m0	n0	t1	NA	g2	NA	NA	1	663
TCGA-CV-7178	64.0	female	stage iva	m0	n1	t4a	21.0	g3	NA	NA	1	2166
TCGA-CV-7180	34.0	male	stage ii	m0	n0	t2	17.0	g2	NA	NA	1	327
TCGA-CV-7183	53.0	male	stage ii	m0	n0	t2	36.0	g1	complete remission/response	no	0	3981

TCGA-CV-7235	67.0	male	stage iii	m0	n0	t3	64.0	g3	complete remission/response	no	0	234 7
TCGA-CV-7236	77.0	female	stage iva	m0	n2c	t2	34.0	g3	NA	NA	1	144
TCGA-CV-7238	69.0	female	stage ii	m0	n0	t2	73.0	g2	complete remission/response	no	0	272 7
TCGA-CV-7242	60.0	female	stage iii	m0	n0	t3	43.0	g2	NA	NA	0	109 5
TCGA-CV-7245	62.0	male	stage iii	m0	n0	t3	46.0	g2	NA	NA	0	797
TCGA-CV-7247	55.0	male	stage ii	m0	n0	t2	9.0	g2	NA	NA	1	577
TCGA-CV-7248	63.0	female	stage iva	m0	n2b	t4a	24.0	g2	NA	NA	1	521
TCGA-CV-7250	64.0	male	stage iii	m0	n0	t3	48.0	g1	complete remission/response	no	1	290 0
TCGA-CV-7252	62.0	female	stage iii	m0	n0	t3	34.0	g2	progressive disease	no	1	151
TCGA-CV-7253	58.0	male	stage ii	m0	n0	t2	NA	g2	partial remission/response	no	1	361
TCGA-CV-7254	55.0	male	stage ii	m0	n0	t2	0.0	g3	NA	NA	1	145 9
TCGA-CV-7255	32.0	female	stage ii	m0	n0	t2	NA	g2	complete remission/response	no	1	64
TCGA-CV-7261	57.0	male	stage iii	m0	n0	t3	17.0	g2	NA	no	0	151 2
TCGA-CV-7263	64.0	male	stage ii	m0	nx	t2	NA	g1	NA	NA	1	560
TCGA-CV-7406	49.0	male	stage ii	m0	n0	t2	2.0	gx	NA	NA	1	174 8

TCGA-CV-7407	67.0	female	stage ii	m0	n0	t2	NA	g2	NA	NA	1	108 1
TCGA-CV-7410	61.0	male	stage ii	m0	n0	t2	10.0	gx	NA	NA	1	641 7
TCGA-CV-7411	64.0	female	stage iva	m0	n0	t4a	29.0	g1	NA	NA	1	271 7
TCGA-CV-7413	74.0	female	stage ii	m0	n0	t2	NA	g2	complete remission/response	no	1	294
TCGA-CV-7414	78.0	male	stage iii	m0	n1	t3	21.0	g2	complete remission/response	no	1	14
TCGA-CV-7415	60.0	male	stage iii	m0	n0	t3	82.0	g2	NA	NA	1	695
TCGA-CV-7416	29.0	female	stage iva	m0	n0	t4a	NA	gx	NA	NA	1	763
TCGA-CV-7418	62.0	male	stage iva	m0	n0	t4a	28.0	g2	complete remission/response	no	1	789
TCGA-CV-7421	76.0	male	stage iva	m0	n0	t4a	11.0	g2	NA	no	1	2
TCGA-CV-7422	60.0	female	stage iva	m0	n1	t4a	27.0	g3	complete remission/response	no	1	103 7
TCGA-CV-7423	65.0	male	stage ii	m0	n0	t2	NA	g1	NA	NA	1	305 9
TCGA-CV-7424	67.0	male	stage iva	m0	n2a	t4a	4.0	g2	NA	NA	1	453
TCGA-CV-7425	77.0	female	stage iii	m0	n0	t3	NA	g1	NA	NA	1	171 8
TCGA-CV-7427	73.0	female	stage ii	m0	n0	t2	NA	g1	complete	no	1	476

									remission/response			0
TCGA-CV-7428	47.0	male	stage iva	m0	n2a	t4a	10.0	g2	NA	NA	1	1671
TCGA-CV-7429	55.0	male	stage iii	m0	n0	t3	24.0	g3	partial remission/response	no	1	107
TCGA-CV-7430	56.0	male	stage iii	m0	n0	t3	10.0	g2	NA	NA	1	495
TCGA-CV-7432	79.0	male	stage iii	m0	n1	t2	20.0	g1	complete remission/response	no	1	2570
TCGA-CV-7433	49.0	male	stage iva	m0	n1	t4a	43.0	g3	progressive disease	yes	1	601
TCGA-CV-7434	64.0	male	stage iii	m0	n0	t3	1.0	g2	complete remission/response	no	1	218
TCGA-CV-7435	57.0	female	stage iva	m0	n1	t4a	22.0	g2	complete remission/response	no	1	4680
TCGA-CV-7437	77.0	male	stage ii	m0	n0	t2	NA	gx	NA	NA	1	506
TCGA-CV-7438	87.0	female	stage ii	m0	n0	t2	NA	g3	NA	no	1	194
TCGA-CV-7440	38.0	male	stage ii	m0	n0	t2	NA	gx	NA	NA	1	675
TCGA-CV-7446	66.0	male	stage ii	m0	n0	t2	2.0	g2	NA	NA	1	1093
TCGA-CV-7568	48.0	female	stage iva	m0	n0	t4a	NA	gx	NA	NA	1	927
TCGA-CV-A45O	57.0	male	stage iii	m0	n0	t3	14.0	g2	NA	no	0	851
TCGA-CV-A45P	82.0	female	stage ii	m0	n0	t2	22.0	g2	complete remission/response	NA	0	639
TCGA-CV-A45Q	69.0	female	stage ivc	m0	n1	t4a	20.0	g1	complete remission/response	no	1	5152

TCGA-CV-A45R	46.0	male	stage iii	m0	n1	t3	26.0	g1	complete remission/response	no	0	5480
TCGA-CV-A45T	64.0	female	stage ii	m0	n0	t2	32.0	g3	NA	no	1	4856
TCGA-CV-A45U	59.0	male	stage iva	m0	n0	t4	13.0	g1	complete remission/response	no	1	1079
TCGA-CV-A45V	87.0	female	stage iva	m0	n0	t4	NA	g1	NA	no	1	32
TCGA-CV-A45W	75.0	male	stage iii	m0	n0	t3	48.0	g2	NA	no	1	1398
TCGA-CV-A45X	47.0	male	stage iva	m0	n1	t4a	32.0	g2	complete remission/response	no	1	198
TCGA-CV-A45Y	61.0	male	stage iva	m0	n0	t4a	15.0	g3	complete remission/response	no	1	2703
TCGA-CV-A45Z	75.0	male	stage ii	m0	n0	t2	10.0	g3	NA	no	1	1466
TCGA-CV-A460	72.0	male	stage iva	m0	n2b	t3	80.0	g3	complete remission/response	no	1	1838
TCGA-CV-A461	65.0	male	stage iii	m0	n0	t3	21.0	g2	complete remission/response	no	1	2064
TCGA-CV-A463	82.0	female	stage iva	m0	n0	t4a	41.0	g2	complete remission/response	no	1	23
TCGA-CV-A464	48.0	male	stage iva	m0	n0	t4a	37.0	g2	complete remission/response	NA	0	1722
TCGA-CV-A465	24.0	male	stage ii	m0	n0	t2	53.0	g1	complete remission/response	no	1	215

TCGA-CV-A468	42.0	male	stage iii	m0	n2b	t3	34.0	g2	complete remission/response	no	1	464
TCGA-CV-A6JD	82.0	female	stage iva	m0	n0	t4a	23.0	g3	complete remission/response	no	1	182
TCGA-CV-A6JE	78.0	male	stage ii	m0	n0	t2	34.0	g2	complete remission/response	no	0	107 5
TCGA-CV-A6JM	85.0	male	stage iva	m0	n0	t4a	38.0	g2	complete remission/response	no	1	194
TCGA-CV-A6JN	53.0	male	stage ii	m0	n0	t2	27.0	g1	complete remission/response	no	0	906
TCGA-CV-A6JO	69.0	male	stage iva	m0	n2c	t4	51.0	g2	progressive disease	NA	1	197
TCGA-CV-A6JT	65.0	male	stage ii	m0	n0	t2	34.0	g2	complete remission/response	no	0	852
TCGA-CV-A6JU	61.0	female	stage iva	m0	n0	t4a	93.0	g2	complete remission/response	NA	0	110
TCGA-CV-A6JY	69.0	male	stage iva	m0	n0	t4a	27.0	g1	complete remission/response	NA	0	646
TCGA-CV-A6JZ	68.0	male	stage iva	m0	n0	t4a	66.0	g2	complete remission/response	no	0	714
TCGA-CV-A6K0	58.0	male	stage ii	m0	n0	t2	20.0	g3	complete remission/response	NA	0	606
TCGA-CV-A6K1	65.0	male	stage iva	m0	n0	t4a	44.0	g2	complete remission/response	no	0	685
TCGA-CV-A6K2	79.0	male	stage ii	m0	n0	t2	49.0	g1	progressive disease	NA	1	317
TCGA-CX-7085	77.0	female	stage iii	m0	n0	t3	25.0	g2	complete	no	0	321

									remission/response			
TCGA-CX-7086	53.0	male	stage iva	m0	n2c	t4a	64.0	g2	complete remission/response	yes	0	573
TCGA-CX-A4AQ	56.0	male	stage iva	m0	n2a	t1	41.0	g3	complete remission/response	NA	0	1555
TCGA-D6-6515	82.0	female	stage ii	m0	n0	t2	NA	g3	complete remission/response	no	1	403
TCGA-D6-6516	69.0	male	stage iii	m0	n1	t1	8.0	g2	complete remission/response	no	0	773
TCGA-D6-6517	59.0	male	stage iii	m0	n0	t3	3.0	g2	progressive disease	yes	0	292
TCGA-D6-6823	50.0	male	stage iii	m0	n1	t3	6.0	g2	complete remission/response	no	0	701
TCGA-D6-6824	61.0	male	stage iva	m0	n1	t4a	25.0	g2	complete remission/response	no	0	77
TCGA-D6-6825	73.0	male	stage iii	m0	n0	t3	9.0	g2	complete remission/response	no	0	491
TCGA-D6-6826	64.0	female	stage iva	m0	n2	t4a	5.0	g2	complete remission/response	no	1	348
TCGA-D6-6827	55.0	female	stage iii	m0	n1	t1	1.0	g3	complete remission/response	no	0	568
TCGA-D6-8568	62.0	male	stage ii	m0	n0	t2	3.0	g2	stable disease	yes	0	759
TCGA-D6-8569	52.0	male	stage ii	m0	n0	t2	7.0	g2	stable disease	no	0	770
TCGA-D6-A4Z9	59.0	male	stage iva	m0	n2	t2	10.0	g2	complete remission/response	no	0	539
TCGA-D6-A4ZB	61.0	male	stage iii	m0	n0	t3	15.0	g2	complete remission/response	no	0	376

TCGA-D6-A6EK	67.0	male	stage iva	m0	n1	t4	3.0	g1	complete remission/response	yes	0	875
TCGA-D6-A6EM	65.0	female	stage iii	m0	n1	t2	17.0	g2	complete remission/response	no	0	232
TCGA-D6-A6EN	71.0	male	stage iii	m0	n0	t3	15.0	g3	complete remission/response	no	0	687
TCGA-D6-A6EO	44.0	male	stage iva	m0	n0	t4a	9.0	g2	stable disease	no	0	759
TCGA-D6-A6EP	62.0	male	stage iii	m0	n0	t3	4.0	g3	complete remission/response	NA	0	424
TCGA-D6-A6EQ	57.0	male	stage iva	m0	n1	t4a	20.0	g3	complete remission/response	NA	0	368
TCGA-D6-A6ES	50.0	male	stage iva	m0	n1	t4a	13.0	g2	complete remission/response	no	0	389
TCGA-D6-A74Q	67.0	male	stage iva	m0	n0	t4a	7.0	g3	complete remission/response	no	0	710
TCGA-DQ-5624	43.0	female	stage iva	m0	n1	t4a	24.0	g2	complete remission/response	yes	0	1778
TCGA-DQ-5625	52.0	female	stage ii	m0	n0	t2	19.0	g2	complete remission/response	no	1	1133
TCGA-DQ-5630	73.0	male	stage iii	m0	n1	t3	49.0	g2	complete remission/response	no	0	1030
TCGA-DQ-5631	52.0	male	stage iva	m0	n2b	t3	20.0	g3	complete remission/response	yes	1	548
TCGA-DQ-7591	62.0	male	stage iva	m0	n2b	t4a	NA	g4	complete remission/response	yes	0	622

TCGA-DQ-7592	57.0	male	stage iva	m0	n2b	t4a	42.0	g2	complete remission/response	yes	0	1143
TCGA-F7-7848	47.0	male	stage iva	m0	n1	t4a	4.0	g2	complete remission/response	no	0	1131
TCGA-F7-8298	58.0	male	stage i	m0	n0	t1	14.0	g1	complete remission/response	no	0	995
TCGA-F7-8489	48.0	male	stage ii	m0	n0	t2	8.0	g1	complete remission/response	no	0	658
TCGA-F7-A50G	66.0	male	NA	NA	NA	NA	9.0	g1	complete remission/response	no	0	616
TCGA-F7-A50I	72.0	male	NA	NA	NA	NA	NA	g2	NA	NA	0	92
TCGA-F7-A50J	67.0	female	NA	NA	NA	NA	10.0	g2	complete remission/response	yes	0	947
TCGA-F7-A61S	62.0	male	NA	mx	nx	tx	6.0	g1	complete remission/response	no	0	576
TCGA-F7-A61V	54.0	male	NA	mx	nx	tx	62.0	g1	complete remission/response	no	0	759
TCGA-F7-A61W	51.0	male	NA	mx	nx	tx	9.0	g2	NA	NA	0	14
TCGA-F7-A620	47.0	male	NA	mx	nx	tx	12.0	g1	complete remission/response	no	0	543
TCGA-F7-A622	75.0	male	NA	mx	nx	tx	NA	g1	complete remission/response	no	1	359
TCGA-F7-A623	70.0	male	NA	mx	nx	tx	6.0	g1	complete remission/response	no	0	616
TCGA-F7-A624	73.0	male	NA	mx	nx	tx	NA	g3	complete remission/response	no	0	378

TCGA-H7-7774	75.0	female	stage iii	m0	n0	t3	21.0	g2	complete remission/response	no	0	407
TCGA-H7-8501	54.0	male	stage iva	m0	n2a	t4a	43.0	gx	complete remission/response	yes	0	461
TCGA-H7-8502	50.0	male	stage iva	m0	n2b	t4a	40.0	g2	complete remission/response	yes	0	458
TCGA-H7-A76A	57.0	male	stage iva	m0	n2b	t2	31.0	g2	complete remission/response	no	0	637
TCGA-HD-7229	60.0	male	stage iva	m0	nx	t4a	NA	g3	progressive disease	yes	0	1027
TCGA-HD-7753	62.0	male	stage ii	m0	n0	t2	NA	g2	complete remission/response	yes	0	866
TCGA-HD-7754	69.0	male	stage iva	m0	n2b	t2	23.0	g1	complete remission/response	NA	0	783
TCGA-HD-7831	74.0	male	stage iii	m0	n0	t3	21.0	g2	complete remission/response	no	0	667
TCGA-HD-7832	52.0	male	stage iva	m0	n2	t4a	35.0	g2	complete remission/response	no	0	836
TCGA-HD-8224	63.0	male	stage iii	m0	n0	t3	57.0	g3	progressive disease	yes	1	446
TCGA-HD-8314	58.0	male	stage iii	m0	n1	t1	44.0	g4	complete remission/response	yes	0	670
TCGA-HD-A4C1	41.0	female	stage iva	m0	n2	t3	42.0	g1	NA	NA	0	11
TCGA-HD-A633	74.0	male	stage iva	m0	n0	t4	39.0	g2	complete remission/response	no	0	421
TCGA-HD-A634	56.0	male	stage iii	m0	n1	t3	22.0	g3	NA	NA	1	130

TCGA-HD-A6HZ	79.0	female	stage ii	m0	n0	t2	11.0	g2	NA	NA	0	111
TCGA-HD-A6I0	56.0	male	stage iii	m0	nx	t3	50.0	g1	complete remission/response	no	0	210
TCGA-HL-7533	65.0	male	stage iva	m0	n2b	t2	NA	g3	complete remission/response	yes	0	1057
TCGA-IQ-7630	49.0	male	stage iii	m0	n0	t3	27.0	g3	complete remission/response	yes	0	485
TCGA-IQ-7631	60.0	female	stage ii	m0	n0	t2	48.0	g1	complete remission/response	no	0	1172
TCGA-IQ-7632	68.0	female	stage iva	m0	n0	t4a	30.0	g1	complete remission/response	no	0	441
TCGA-IQ-A61E	55.0	female	stage iii	mx	n0	t3	44.0	g2	complete remission/response	yes	0	1147
TCGA-IQ-A61G	57.0	male	stage iva	mx	n2c	t4a	39.0	g2	progressive disease	yes	0	360
TCGA-IQ-A61H	76.0	male	stage ii	mx	n0	t2	53.0	g2	complete remission/response	no	0	1138
TCGA-IQ-A61I	63.0	male	stage iva	m0	n2a	t1	41.0	g3	complete remission/response	NA	1	2
TCGA-IQ-A61J	54.0	male	stage iva	m0	n2b	t2	73.0	g1	complete remission/response	yes	0	1021
TCGA-IQ-A61O	43.0	male	stage iva	mx	n2b	t2	38.0	gx	complete remission/response	yes	1	421
TCGA-IQ-A6SH	55.0	male	stage ivc	m1	n1	t2	49.0	g1	complete remission/response	no	0	471
TCGA-KU-A66S	69.0	female	stage iii	mx	n1	t2	7.0	g2	complete	no	1	406

									remission/response			
TCGA-KU-A66T	53.0	female	stage iva	m0	n0	t4	24.0	g2	complete remission/response	no	0	552
TCGA-KU-A6H7	55.0	female	stage iva	m0	n2a	t2	23.0	g2	complete remission/response	yes	0	586
TCGA-KU-A6H8	41.0	male	stage i	m0	n0	t1	16.0	g3	progressive disease	yes	1	327
TCGA-MT-A51W	52.0	female	stage i	m0	nx	t1	NA	g2	complete remission/response	no	0	437
TCGA-MT-A51X	30.0	male	stage iva	m0	n2b	t1	35.0	g1	NA	no	0	242
TCGA-MT-A67A	85.0	female	stage i	m0	n0	t1	20.0	g2	complete remission/response	no	0	914
TCGA-MT-A67D	55.0	male	stage ii	m0	n0	t2	23.0	g2	complete remission/response	NA	0	56
TCGA-MT-A67F	60.0	female	stage iva	mx	n0	t4	64.0	g2	stable disease	yes	0	384
TCGA-MT-A7BN	74.0	male	stage iva	m0	n2b	t2	19.0	g3	progressive disease	yes	0	469
TCGA-MZ-A5BI	53.0	male	stage iva	m0	n2b	t2	NA	NA	complete remission/response	NA	1	217
TCGA-MZ-A6I9	68.0	male	stage iva	m0	n2b	t4a	NA	g2	NA	no	1	489
TCGA-MZ-A7D7	51.0	male	stage iva	m0	n2b	t3	72.0	NA	complete remission/response	yes	0	547
TCGA-P3-A5Q5	54.0	male	stage iva	m0	n2	tx	NA	g3	complete remission/response	NA	0	910

TCGA-P3-A5Q6	49.0	male	stage iii	m0	n0	t3	35.0	g3	complete remission/response	yes	1	480
TCGA-P3-A5QA	41.0	male	stage ii	m0	n0	t2	NA	g3	complete remission/response	no	0	2182
TCGA-P3-A5QE	49.0	male	stage iii	m0	n1	t1	17.0	g2	complete remission/response	no	0	1559
TCGA-P3-A5QF	49.0	male	stage iva	m0	n2b	t4	41.0	g2	NA	NA	1	330
TCGA-P3-A6SW	50.0	male	stage iva	m0	n2b	t4	NA	g3	complete remission/response	NA	0	1120
TCGA-P3-A6SX	67.0	male	stage iva	m0	n0	t4	30.0	g2	complete remission/response	NA	1	1430
TCGA-P3-A6T0	47.0	female	stage iva	m0	n2b	t4	18.0	g2	complete remission/response	NA	0	578
TCGA-P3-A6T2	45.0	male	stage ii	m0	n0	t2	42.0	g2	complete remission/response	yes	0	2298
TCGA-P3-A6T3	49.0	male	stage iva	m0	n1	t4	30.0	g2	complete remission/response	NA	1	577
TCGA-P3-A6T4	54.0	male	stage iva	m0	n1	t4	14.0	g2	progressive disease	NA	1	62
TCGA-P3-A6T5	79.0	female	stage iva	m0	n0	t4	29.0	g2	complete remission/response	no	1	882
TCGA-P3-A6T6	53.0	male	stage iva	m0	n0	t4	NA	g3	complete remission/response	no	1	395
TCGA-P3-A6T7	55.0	male	stage iii	m0	n1	t3	47.0	g2	complete remission/response	NA	1	487

TCGA-P3-A6T8	54.0	male	stage iva	m0	n0	t4a	34.0	g3	complete remission/response	NA	0	400
TCGA-QK-A64Z	79.0	female	stage ii	m0	n0	t2	NA	g1	progressive disease	no	1	641
TCGA-QK-A652	60.0	male	stage ii	m0	n0	t2	39.0	g2	complete remission/response	no	0	645
TCGA-QK-A6IF	61.0	male	stage iva	m0	n2b	t1	NA	gx	complete remission/response	yes	0	704
TCGA-QK-A6IG	69.0	male	stage ii	m0	n0	t2	22.0	g2	progressive disease	yes	1	222
TCGA-QK-A6IH	65.0	female	stage iva	m0	n1	t4a	32.0	g2	progressive disease	yes	0	653
TCGA-QK-A6II	52.0	male	stage iii	m0	n0	t3	58.0	g2	progressive disease	yes	1	284
TCGA-QK-A6IJ	71.0	male	stage ii	m0	n0	t2	40.0	g3	NA	no	0	387
TCGA-QK-A6V9	56.0	male	stage ii	m0	n0	t2	NA	g2	complete remission/response	no	0	833
TCGA-QK-A6VB	66.0	male	stage iva	m0	n0	t4a	40.0	g2	complete remission/response	no	0	641
TCGA-QK-A6VC	62.0	female	stage iva	m0	n2c	t4a	67.0	g2	complete remission/response	no	0	600
TCGA-QK-A8Z7	59.0	male	stage iva	m0	n2c	t4a	96.0	NA	complete remission/response	yes	0	392
TCGA-QK-A8Z8	60.0	female	stage ivc	m1	n1	t3	NA	g2	NA	NA	1	171
TCGA-QK-A8Z9	56.0	male	stage iva	m0	n2b	t4a	82.0	g2	complete remission/response	yes	1	449
TCGA-QK-A8ZA	60.0	male	stage iva	mx	n2b	t2	NA	g2	complete remission/response	yes	1	371

TCGA-QK-A8ZB	68.0	male	stage iva	m0	n0	t4a	81.0	g2	complete remission/response	no	0	542
TCGA-QK-AA3J	69.0	male	stage i	m0	n0	t1	NA	g3	complete remission/response	no	0	466
TCGA-QK-AA3K	60.0	male	stage iva	mx	n2b	t3	6.0	g2	NA	yes	0	253
TCGA-RS-A6TO	82.0	female	stage iva	m0	n2c	t4	61.0	g2	complete remission/response	yes	1	387
TCGA-RS-A6TP	58.0	male	stage ii	m0	n0	t2	NA	g3	complete remission/response	no	0	516
TCGA-T2-A6WX	73.0	female	stage iii	m0	n0	t3	85.0	g1	progressive disease	NA	1	209
TCGA-T2-A6WZ	53.0	male	stage iva	m0	n2b	t3	89.0	g2	stable disease	yes	1	484
TCGA-T2-A6X0	49.0	male	stage iva	m0	n2a	t1	31.0	g2	complete remission/response	NA	0	216
TCGA-T2-A6X2	82.0	male	stage iii	m0	n0	t3	53.0	g1	complete remission/response	NA	0	987
TCGA-T3-A92M	52.0	male	stage iva	m0	n2c	t4a	4.0	g2	NA	yes	0	417
TCGA-T3-A92N	79.0	male	stage ivc	m1	n1	t1	31.0	g3	progressive disease	no	1	95
TCGA-TN-A7HI	56.0	male	stage i	m0	n0	t1	49.0	g3	complete remission/response	no	0	412
TCGA-TN-A7HJ	51.0	male	stage iii	m0	nx	t3	121.0	g2	complete remission/response	yes	0	403
TCGA-TN-A7HL	59.0	male	stage	m0	nx	t4a	71.0	g2	complete	yes	0	619

			iva						remission/response			
TCGA-UF-A718	62.0	male	stage iva	m0	n0	t4a	25.0	g2	complete remission/response	NA	0	197 1
TCGA-UF-A719	54.0	male	stage iii	m0	n0	t3	13.0	g1	complete remission/response	no	0	166 3
TCGA-UF-A71A	67.0	male	stage iva	m0	n2a	t3	31.0	g1	complete remission/response	NA	1	86
TCGA-UF-A71B	50.0	male	stage iva	m0	n1	t4a	11.0	g2	complete remission/response	no	0	150 6
TCGA-UF-A71E	63.0	male	stage iva	m0	n0	t4	48.0	g3	complete remission/response	no	1	150 4
TCGA-UF-A7J9	75.0	male	stage iva	m0	n0	t4a	18.0	g2	complete remission/response	no	0	135 8
TCGA-UF-A7JA	66.0	female	stage iva	m0	n1	t4a	14.0	g2	complete remission/response	no	0	226 5
TCGA-UF-A7JC	42.0	male	stage iii	m0	n1	t3	37.0	g1	complete remission/response	yes	1	546
TCGA-UF-A7JD	71.0	male	stage iva	m0	n0	t4a	25.0	g3	complete remission/response	no	1	739
TCGA-UF-A7JF	80.0	male	stage iva	m0	n2b	t4a	66.0	g2	complete remission/response	yes	0	168 6
TCGA-UF-A7JH	59.0	male	stage iva	m0	n0	t4a	24.0	g1	complete remission/response	no	0	896
TCGA-UF-A7JJ	68.0	male	stage iva	m0	n0	t4a	27.0	g1	complete remission/response	yes	0	549
TCGA-UF-A7JK	59.0	male	stage	m0	n0	t4a	19.0	g2	complete	NA	1	424

			iva						remission/response			
TCGA-UF-A7JO	79.0	female	stage iva	m0	n0	t4a	27.0	g2	complete remission/response	NA	1	631
TCGA-UF-A7JS	59.0	male	stage iva	m0	n2b	t3	54.0	g2	complete remission/response	no	1	680
TCGA-UF-A7JT	72.0	female	stage iva	m0	n0	t4a	13.0	g3	complete remission/response	NA	1	993
TCGA-UP-A6WW	58.0	male	stage iva	mx	n2c	t2	53.0	g2	complete remission/response	no	0	518
TCGA-WA-A7H4	69.0	male	stage ii	m0	n0	t2	NA	g3	complete remission/response	no	0	443
